# Intramyocardial Sprouting Tip Cells Specify Coronary Arterialization

**DOI:** 10.1161/CIRCRESAHA.124.324868

**Published:** 2024-08-14

**Authors:** Elena Cano, Jennifer Schwarzkopf, Masatoshi Kanda, Eric L. Lindberg, Irene Hollfinger, Cristina Pogontke, Caroline Braeuning, Cornelius Fischer, Norbert Hübner, Holger Gerhardt

**Affiliations:** Integrative Vascular Biology Laboratory (E.C., J.S., I.H., H.G.), Max-Delbrück Center for Molecular Medicine in the Helmholtz Association (MDC), Berlin, Germany.; Cardiovascular and Metabolic Sciences (M.K., E.L.L., N.H.), Max-Delbrück Center for Molecular Medicine in the Helmholtz Association (MDC), Berlin, Germany.; DZHK (German Center for Cardiovascular Research), Berlin, Germany (E.C., J.S., N.H., H.G.).; Charité-Universitätsmedizin, Berlin, Germany (E.C., J.S., N.H., H.G.).; Department of Animal Biology, University of Málaga, Spain (E.C., C.P.).; Cardiovascular Development and Disease, Biomedical Research Institute of Malaga and Nanomedicine Platform (IBIMA - BIONAND Platform), Málaga, Spain (E.C., C.P.).; Department of Rheumatology and Clinical Immunology, Sapporo Medical University, Japan (M.K.).; Department of Medicine, Ludwig-Maximiliams-University Munich, Germany (E.L.L.).; BIH/MDC Genomics Technology Platform, Berlin, Germany (C.B., C.F.).; Berlin Institute of Health (BIH), Germany (H.G.).

**Keywords:** angiogenesis, arteries, coronary vessels, endocardium, endothelium

## Abstract

**BACKGROUND:**

The elaborate patterning of coronary arteries critically supports the high metabolic activity of the beating heart. How coronary endothelial cells coordinate hierarchical vascular remodeling and achieve arteriovenous specification remains largely unknown. Understanding the molecular and cellular cues that pattern coronary arteries is crucial to develop innovative therapeutic strategies that restore functional perfusion within the ischemic heart.

**METHODS:**

Single-cell transcriptomics and histological validation were used to delineate heterogeneous transcriptional states of the developing and mature coronary endothelium with a focus on sprouting endothelium and arterial cell specification. Genetic lineage tracing and high-resolution 3-dimensional imaging were used to characterize the origin and mechanisms of coronary angiogenic sprouting, as well as to fate-map selective endothelial lineages. Integration of single-cell transcriptomic data from ischemic adult mouse hearts and human embryonic data served to assess the conservation of transcriptional states across development, disease, and species.

**RESULTS:**

We discover that coronary arteries originate from cells that have previously transitioned through a specific tip cell phenotype. We identify nonoverlapping intramyocardial and subepicardial tip cell populations with differential gene expression profiles and regulatory pathways. *Esm1*-lineage tracing confirmed that intramyocardial tip cells selectively contribute to coronary arteries and endocardial tunnels, but not veins. Notably, prearterial cells are detected from development stages to adulthood, increasingly in response to ischemic injury, and in human embryos, suggesting that tip cell-to-artery specification is a conserved mechanism.

**CONCLUSIONS:**

A tip cell-to-artery specification mechanism drives arterialization of the intramyocardial plexus and endocardial tunnels throughout life and is reactivated upon ischemic injury. Differential sprouting programs govern the formation and specification of the venous and arterial coronary plexus.

**Novelty and Significance**

**What Is Known?**

- The connection of coronary plexus to aortic root initiates blood flow onset, which triggers coronary plexus arterialization.
- Cardiac prearterial cells are flow-independent specified precursors that contribute to coronary artery assembly.
- Cardiac prearterial cells specification requires cell cycle suppression.

**What New Information Does This Article Contribute?**

- Cardiac prearterial cells are specified during tip cell sprouting toward the myocardium.
- Intramyocardial and subepicardial tip cells exhibit different transcriptional signatures and regulatory signaling.
- Tip cell-to-artery specification contributes to the formation of coronary plexus and endocardial tunnel arterialization.
- Prearterial cells show a remarkably persistent transcriptional profile from development to adulthood in mouse and are conserved in human embryonic hearts.
- Prearterial cell population increases in response to ischemic injury.

Cardiac ischemia, resulting from inadequate blood supply to the heart muscle, remains a leading cause of morbidity and mortality worldwide. The formation of functional blood vessels within the ischemic area is critically important for the restoration of cardiac function and the prognosis of the patient. Up to now, the attempted revascularization strategies have been ineffective in establishing a stable, mature vascular network capable of providing functional perfusion, thus resulting in a failure to improve cardiac function. The lack of deeper understanding of the intricate molecular, cellular, and structural cues essential for the formation of a hierarchical blood vessel network represents an obstacle to the development of effective experimental therapies for the treatment of cardiac disease. The developmental program that orchestrates the arteriovenous specification of coronary vessels provides a framework to investigate this process. This study aims to investigate the mechanisms of coronary arterialization with a particular focus on the origin and characterization of the recently described prearterial cells. Our findings identify tip cell-to-artery specification as a mechanism of coronary plexus arterialization and highlight its persistence throughout life, as well as its reactivation following myocardial infarction. Furthermore, we demonstrate its significance in human heart development, therefore, suggesting potential future avenues for therapeutic intervention in cardiac ischemia.

**Meet the First Author, see p 637**

The general model of vascular remodeling, describing the reorganization of a primordial network of capillaries into a hierarchical network of functional arteries and veins, sustains that this process is elicited by blood flow.^1^ Blood circulation exerts mechanical forces on endothelial cells (ECs), triggering coordinated changes at the single-cell level, such as in polarization, shape, migration, and proliferation rates. Flow-mediated shear stress also induces Notch signaling, setting off downstream signaling cascades that culminate in arterial specification.^2,3^ As a counterplayer, the transcription factor Nr2f2 (nuclear receptor subfamily 2 group F member 2; CouptfII) negatively regulates Notch and thereby promotes venous specification^4^ also in human ECs.^5^ Likewise, in the heart, arteriovenous specification of the coronary plexus is widely accepted to be triggered by the onset of effective blood flow, occurring when the coronary plexus establishes a connection to the root of the aorta.^6–8^ This embryonic event initiates the specification of superficial (subepicardial) coronary veins and deeper (intramyocardial) coronary arteries (Figure 1A). Indeed, remodeling of the coronary vasculature is defective in mutants in which the coronary vasculature fails to connect to the aorta.^7,9,10^

**Figure 1.**
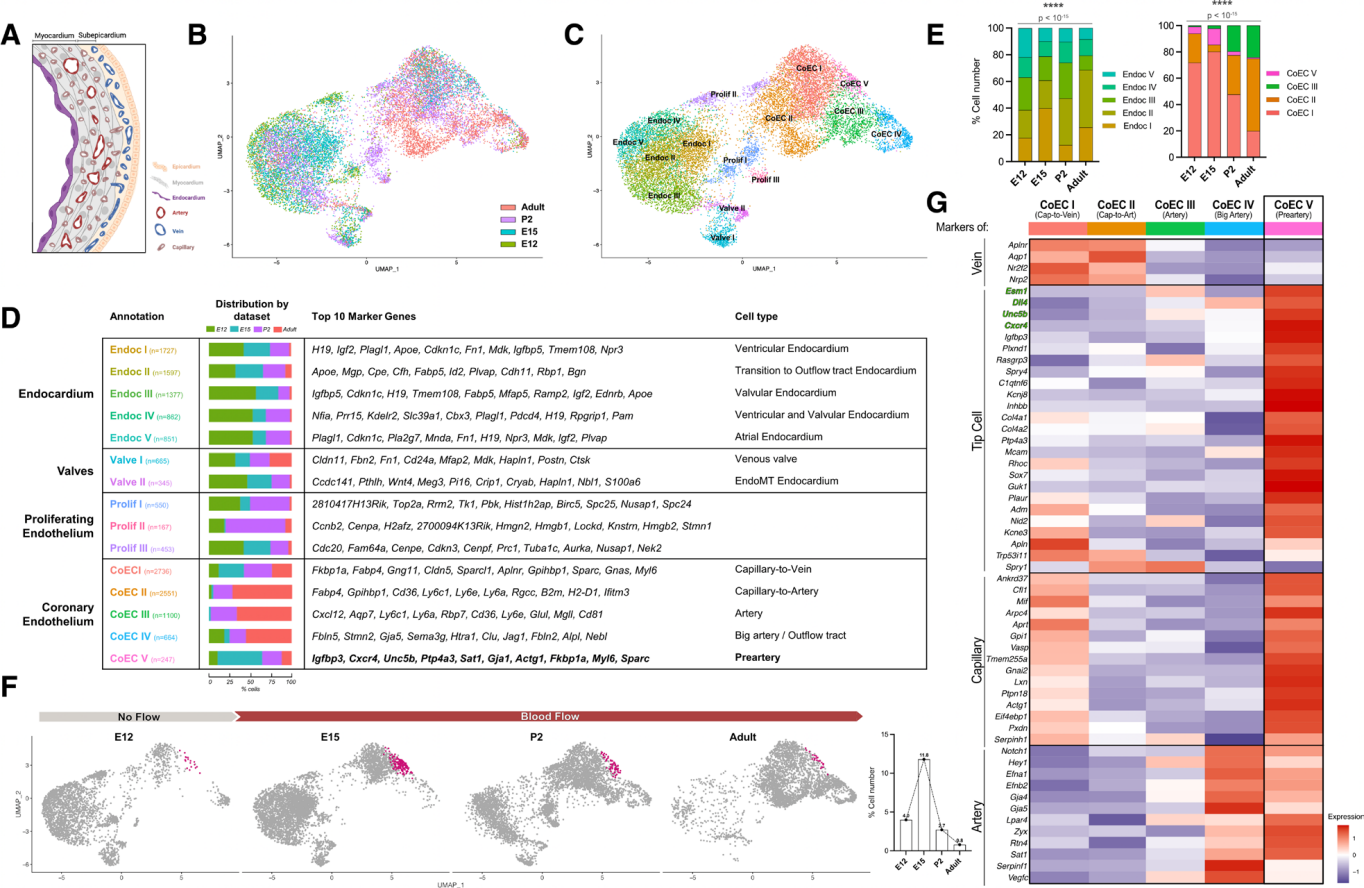
**Prearterial cell population with tip cell signature is detected from development to adulthood. A**, Schematic section of a ventricular wall showing intramyocardial arteries (red) and capillaries (Cap; brown) and subepicardial veins (blue). Endocardial cells (purple) line the luminal side of the chamber. **B**, Uniform manifold approximation and projection (UMAP) plot showing cardiac endothelial cells (ECs) clustering of 4 embryonic stages (E12, E15, postnatal stage P2, and 8-week-old adult hearts) from single-cell sequencing and color-coded by the data set of origin (ie, developmental stages). **C**, UMAP plot of sorted cardiac ECs from E12, E15, P2, and 8-week-old adult hearts, color-coded by cluster. Four groups are identified, each composed of various clusters: coronary endothelium (CoEC I to CoEC V), endocardium (Endoc I to Endoc V), valvular endocardium (valves I and II), and proliferating cells (Prolif I to III). **D**, Overview of annotated clusters. The table shows annotation and cell number (n) of every cluster (first row), cluster distribution throughout the different developmental stages (second row), top 10 marker genes (third row), and the assigned cell type after marker gene validation (last row and Figure S6). **E**, Composition (%) of the endocardium (**left**) and coronary plexus (excluding CoEC IV cluster; **right**) at every studied developmental stage. The distribution of all endocardial clusters (Endoc I to V) is stable over time. The coronary endothelium diversifies over time mainly due to arterialization: CoEC II (capillary-to-artery) and CoEC III (artery) become predominant after birth. The percentage is calculated by cell number of every cluster, normalized to total cell number, *P* value by χ^2^ test. **F**, UMAP plot showing CoEC V/preartery cluster highlighted in pink and its quantification at every studied developmental stage. CoEC V/preartery cells are detected before the onset of coronary flow (E12) and remain throughout embryonic, perinatal, and adult life (E15, P2, and 8-week-old adult), with a peak at E15. The graph shows distribution (%) over time of CoEC V cluster normalized to total cell number, as also shown in **E**. **G**, Heatmap of a wide list of known venous, tip cell, capillary, and arterial markers in all coronary EC clusters (CoEC I to CoEC V). CoEC V/preartery cluster shows high expression of capillary, tip cell, and arterial markers.

However, the identification of prearterial cells in zebrafish and mouse retina vasculature,^11–13^ and more recently in the heart,^14–16^ has unveiled the existence of alternative arterialization mechanisms. While in the retina, prearterial specification was described to be preceded by the acquisition of the tip cell position at the sprouting front^11–13^; in the heart, cardiac prearterial cells were defined as individual capillary ECs in the immature plexus that undergo a sudden upregulation of mature artery markers, such as Cx40 (connexin 40), even before the establishment of the coronary blood circulation.^14^ Genetic lineage tracing demonstrated that these Cx40-expressing capillary ECs ultimately incorporate into coronary arteries, a process initiated by the suppression of Nr2f2,^14^ which induces cell cycle arrest.^4,11^ Cell cycle exit was indeed later demonstrated to be the direct regulator of arterialization from prearterial capillaries.^16^ Activation of Notch signaling in capillary ECs leads to the suppression of Myc and, therefore, cell cycle arrest, priming capillary ECs for mobilization and artery assembly.^16^ However, despite these advances, the origin and mechanisms underlying the specification of cardiac prearterial cells remains largely unknown.

Here, we used single-cell transcriptomics in conjunction with corresponding histological validation of candidate markers, to comprehensively unravel the transcriptional states and depict a spatiotemporal map of cardiac prearterial cells throughout embryonic and postnatal life, as well as upon ischemic injury. Notably, prearterial cells represent a remarkably persistent population, with an intermediate transcriptional profile of capillary and arterial cells, and enrichment for tip cell markers, such as *Esm1*. Three-dimensional lineage tracing of *Esm1*-expressing tip cells confirmed that intramyocardial tip cells eventually incorporate into coronary arteries and endocardial tunnels,^17^ suggesting that prearterial cells are specified during intramyocardial sprouting. We discover molecular profiles that characterize distinct sprouting tip cell populations in the intramyocardial and subepicardial layers, shaping prospective coronary arteries and veins, respectively. Together, our data suggest the existence of distinct tip cell subpopulations defined by differential sprouting regulatory mechanisms in the heart, which may play a role in determining the arteriovenous fate. The observation of an increasing prearterial cell population in response to ischemic injury, as well as the identification of the prearterial gene transcriptional signature in human embryonic hearts, suggests that tip cell-to-artery specification is a conserved mechanism across species, which may play a role in the response to myocardial infarction (MI).

Understanding the origins and developmental processes of coronary arteries and veins, including the involvement of prearterial cells, holds promise for designing effective revascularization strategies to deal with ischemic cardiomyopathies.

## METHODS

### Data Availability

Data supporting the findings in this study are included in the main article and associated files. Source data and the complete lists of differentially expressed genes (DEGs) of annotated clusters are provided in this article (Tables S1 and S2). The single-cell RNA sequencing data sets generated and analyzed during the current study are available in the Gene Expression Omnibus using the accession identifier GSE223266.

### Mouse Strains and Tamoxifen-Induced Lineage Tracing

The following mouse strains were used: *PdgfbCreERT* (Tg[Pdgfb-icre/ERT2,-EGFP]1Frut),^18^ *BmxCreERT* (Tg[Bmx-cre/ERT2]1Rha),^19^ *Esm1CreERT2* (Tg[Esm1-cre/ERT2]1Rha),^20^ and *R26mTmG* (Gt[ROSA]26Sortm4[ACTB-tdTomato,-EGFP]Luo/J).^21^ Mice were maintained at the Max-Delbrück Center for Molecular Medicine under standard husbandry conditions and were handled in compliance with the institutional and European Union guidelines for animal care and welfare.

All embryos were staged from the time point of vaginal plug, which was designated as E0. Single injections of tamoxifen (Sigma-Aldrich) were performed intraperitoneally (100 µg/g animal) 24 hours prior analyzed time point for *PdgfbCreERT;R26mTmG*, at E9.5 for *BmxCreERT;R26mTmG*, at E12.5 for *Esm1CreERT;R26mTmG*. Embryos/pups were then collected from E10.5 to P0. Animal procedures were performed in compliance with the European guidelines for animal care and welfare and approved by the corresponding Spanish or German Ethics Committee for Animal Research.

### Statistical Analysis

No statistical methods were used to predetermine the sample size. Sample sizes were chosen based on experience while complying with animal welfare and ethical permit constraints. For imaging, samples were collected from at least 3 independent experiments; no sample randomization was performed as it was not relevant to our correlational study. The images shown in the figures are representative of the corresponding quantification.

For single-cell RNA sequencing, multiple samples were pooled, and no replicate was performed. The number of samples pooled (n) is provided in the Methods section. Batch effects were assessed by grouping cells by the sex of the donor for every stage. No differences were found in the composition or distribution of annotated cell types. No blinding was carried out as it was not relevant to our study.

Graphs and statistical analyses were performed with GraphPad Prism version 9.3.1. When comparing 2 groups of measurements, nonparametric Wilcoxom rank-sum or Mann-Whitney *U* tests were performed, depending on the independence of the groups. When comparing 3 or more groups, Friedman followed by Benjamini and Hochberg multiple comparison test or Kruskal-Wallis test followed by Dunn multiple comparisons test was applied. To check proportions, a χ^2^ test was performed. Each statistical test and its corresponding *P* value are detailed in the corresponding figure and figure legend; significant differences were considered if *P*<0.05. Independent biological replicates (n) are provided in the figure legends. Data are represented as mean±SD with individual data points. No data were excluded from the study.

## RESULTS

### Single-Cell Sequencing Identifies a Persistent Prearterial Cell Population Throughout Life

To unravel the transcriptional profile of coronary prearterial cells and to investigate whether this cellular subtype represents an exclusive transitory state during mouse heart development, we performed single-cell RNA sequencing on fluorescent-activated cell (FAC)-sorted cardiac endothelial fractions (CD31+/CD45−) at 4 different stages: E12.0, E15.0, postnatal day (P)2 and adult (8 weeks old; Figure 1B; Figure S1A). Rigorous quality control was performed, and no sex batch effect was detected. Non-ECs, immediate-early gene-expressing ECs, and lymphatic ECs were excluded from the analysis (see Methods). UMAP (unsupervised clustering using uniform manifold approximation and projection) method on the combined data sets resulted in 4 major groups of clusters, designated as endocardium, coronary endothelium, proliferating cells, and valvular endocardium, based on the expression of the top DEGs (Figure 1C and 1D; Figure S2A). All clusters were annotated upon spatial validation of DEGs using the in situ hybridization GenePaint database^22^ (Figure S2B). The distribution of all observed cell types recapitulated the expected composition of the cardiac tissue. During embryonic stages, the endocardial fraction and proliferative endothelium were predominant, while the coronary endothelium (CoEC) fraction became prevalent in postnatal stages (Figure 1D, second column, Figure S2C).

Within the CoECs population, we identified (1) CoEC I, a microvascular subtype displaying a capillary-to-venous transcriptional profile, (2) CoEC II, a microvascular subtype with a capillary-to-artery profile; (3) CoEC III, representing arterial cells; and (4) CoEC IV, representing large caliber arterial cells, likely including the pulmonary artery and aorta at the outflow tract; and (5) CoEC V, a cluster, which matched the recently identified coronary prearterial transcriptional signature (Figure S3A).^14^ While the distribution of all endocardial clusters remained stable across all stages studied, CoECs showed an increasing diversity from development to adulthood, mainly due to arterialization of the vascular plexus (Figure 1E).

The CoEC V/preartery cluster emerged as early as E12, when the coronary plexus is not yet perfused, representing 4% of total CoECs. This population reached a peak at E15 when they constituted almost 12% of total CoECs. Interestingly, although their number dropped to 1% to 2% of total CoECs in postnatal stages, we could still identify prearterial cells in adult hearts (Figure 1F).

Our data show that prearterial cells exhibit a persistent transcriptional profile throughout embryonic, postnatal and adult stages. Together, our results confirm that prearterial specification is indeed independent of flow^14^ and provide first evidence for the persistence of the prearterial specification mechanism in the adult heart, even after the full patterning of the coronary arterial tree.

### Prearterial Cells Exhibit a Tip Cell Signature

Further examination of the transcriptional profile of the CoEC V/preartery cluster revealed a combination of typical capillary markers, similar to the CoEC I capillary-to-vein cluster and multiple arterial markers, similar to CoEC III and CoEC IV arterial clusters (Figure 1G; Figure S3B). Intriguingly, CoEC V/preartery cells also displayed a significant enrichment of tip cell markers, such as *Cxcr4*,^23^ *Unc5b*,^24^ *Esm1*,^20^ *Dll4*,^25^ and *Igfbp3*^26^ (Figure 1G; Figure S3B).

Analysis of the annotated CoEC V/preartery cells across individual time points (E12, E15, P2, and adult) confirmed their consistent enrichment of tip cell markers (Figure S3C and S3D). Similarly, compared with surrounding capillary cells, they also showed a downregulation of the venous marker *Nr2f2* (Figure S3B and S3D), aligning with previous description of cardiac prearterial cells.^14^

Gene set–enrichment analysis comparing CoEC V/preartery cells to the other CoEC clusters (CoEC I to CoEC IV) highlighted angiogenic and migratory signatures as relevant biological functions (Figure S4A and S4D), with enrichment of Rho GTPases effectors and hypoxic and glycolytic pathways (Figure S4B, S4C, S4E, and S4F). Notably, cell cycle scoring indicated CoEC V/preartery cells exhibit reduced proliferation rates, similar to the levels exhibited by fully specified arterial cells (Figure S4G). We identified a number of upregulated targets directly controlled by p53 and Rb1 transcription factors (Figure S4G), well-known regulators of cell cycle suppressor genes, and which are induced by hypoxia.^27^ Blood flow–induced genes such as *Klf2*, *Klf4*, and *Thbd* were downregulated in comparison to other CoECs (Figure S4H), further supporting the notion that blood flow does not trigger their specification.^14^

In summary, the enrichment of tip cell markers, highly migratory profile, reduced proliferation rate, and expression of genes related to glycolytic metabolism and hypoxia response, collectively suggest that prearterial cells are closely related to tip cells.^28–32^ This is the first time that prearterial cells are identified as sprouting tip cells in the heart.

### Tip Cells Sprout From the Subepicardial Plexus and From the Endocardium

**Figure 2.**
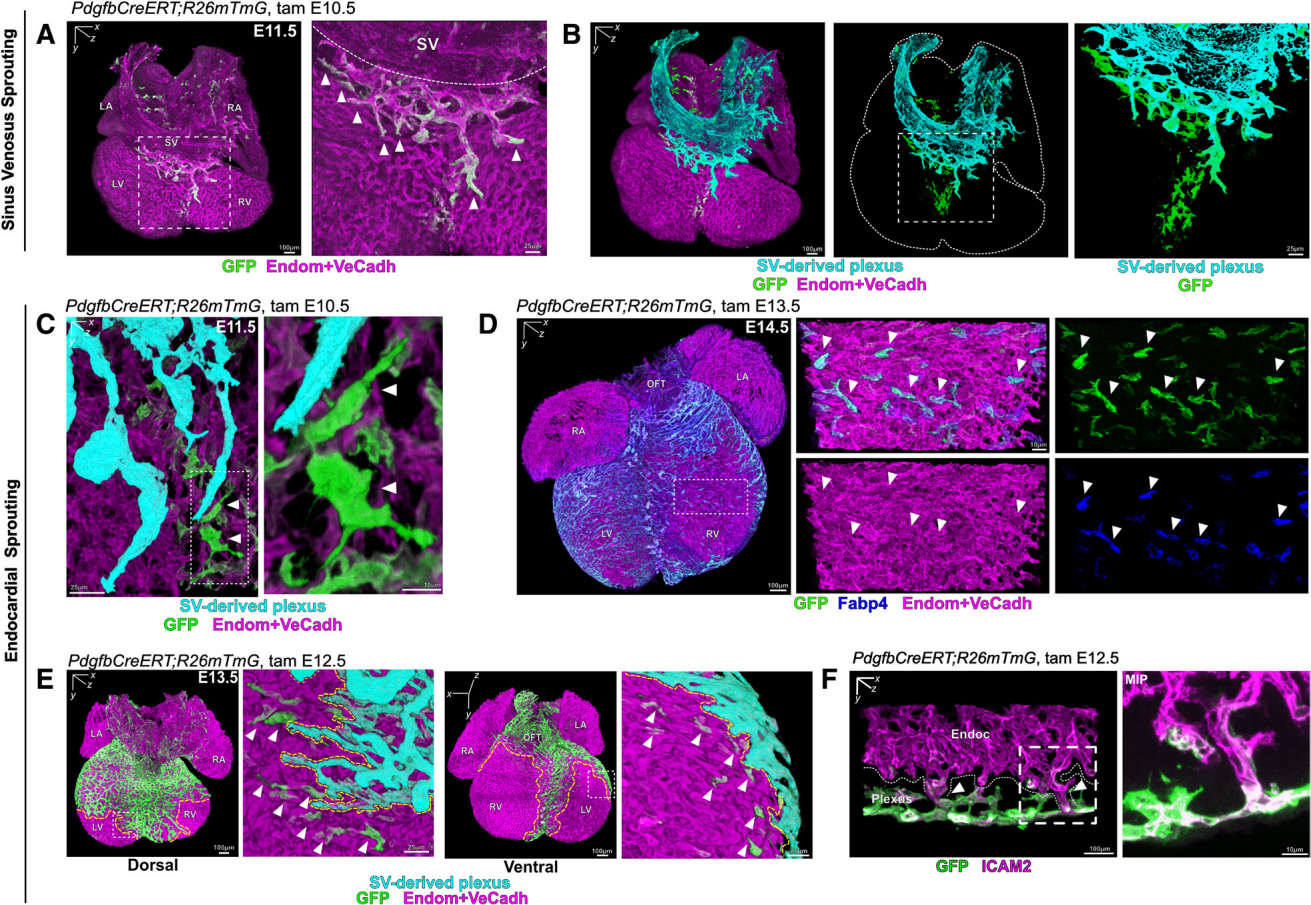
**Sprouting from sinus venosus and endocardium contributes to plexus of free ventricular walls. A**, Three-dimensional rendering of an E11.5 *PdgfbCreERT;R26mTmG* heart. *Pdgfb*-expressing tip cells are labeled by green fluorescent protein (GFP) (green) while all cardiac endothelial cells (ECs; coronary ECs and endocardial) are labeled with a cocktail of anti-endomucin (Endom) and anti-VeCadherin (VeCadh) antibodies (magenta). Please note that *Pdgfb*-expressing cells are also expressing Endom and VeCadh. The boxed area shows sprouting plexus from sinus venosus (SV) and arrowheads point to *Pdgfb*-expressing tip cells at the sprouting front. **B**, Three-dimensional rendering of an E11.5 *PdgfbCreERT;R26mTmG* heart. The SV and the contiguous SV-derived subepicardial plexus are highlighted in cyan by image postprocessing segmentation (see Methods). A significant number of *Pdgfb*-expressing tip cells are found in the vicinity of the subepicardial plexus, still integrated within the endocardium (green cells). **C**, Three-dimensional rendering of the sprouting front of the subepicardial plexus (cyan) of an E11.5 *PdgfbCreERT;R26mTmG* heart. *Pdgfb*-expressing endocardial cells (arrowheads) show a tip cell phenotype, with numerous filopodia, and their longitudinal axis perpendicular to the endocardial lining, protruding toward the myocardium. **D**, Three-dimensional rendering of an E14.5 *PdgfbCreERT;R26mTmG* heart stained against Fabp4 (blue) and Endom and VeCadh (magenta). Sprouting *Pdgfb*-expressing endocardial cells (arrowheads) also express the coronary endothelial marker Fabp4. **E**, Three-dimensional rendering of an E13.5 *PdgfbCreERT;R26mTmG* heart. Boxed areas show a magnification of the SV-derived sprouting front at the dorsal (**left**) and ventral (**right**) aspects of the same heart. *Pdgfb*-expressing endocardial tip cells are in the vicinity of the SV-derived sprouting front (arrowheads). **F**, Three-dimensional rendering of a ventricular wall of an E13.5 *PdgfbCreERT;R26mTmG* heart stained against ICAM2 (intercellular adhesion molecule 2) (magenta). Arrowheads point to lumenized connections between endocardium (magenta) and *Pdgfb*-expressing coronary plexus (green). **Right**, Shows maximum intensity projections (MIPs) of boxed area. LA indicates left atrium; LV, left ventricle; OFT, outflow tract; RA, right atrium; RV, right ventricle; SV, sinus venosus; and Tam, tamoxifen.

Given the tip cell profile of CoEC V/preartery cells, we next sought to characterize potential sprouting events during coronary development. Since *Pdgfb* is well known as a tip cell marker,^25,33,34^ we utilized the *PdgfbCreERT* transgenic mouse strain,^18^ in combination with the *R26mTmG* reporter strain.^19^ Tamoxifen induction performed 24 hours before every time point analyzed served to recapitulate the *Pdgfb* expression pattern of the coronary endothelium (Figure S1A). At the earliest stages of coronary sprouting (E11.5), *Pdgfb* expression revealed a tip cell specific pattern (Figure 2A), expanding to all coronary ECs from E12.5 onward (Figure S5A; Videos S1 through S5).

Consistent with previous observations,^35–40^ we confirmed 2 distinct origins where coronary sprouting is initiated, from the sinus venosus (SV), at the dorsal aspect of the heart (Figure S5A, top row); and from the endocardium, at the ventral aspect of the heart (Figure S5A, bottom row; Videos S1 through S5). While SV-derived vessels populate the outer ventricular walls by inward sprouting (Figure S5B, red arrow), the endocardium vascularizes the interventricular septum and ventral ventricular walls by outward sprouting (purple arrow in Figure S5B, S5C, and S5E; Video S6). Frequently, where myocardial thickness is still low, endocardial cells can be found sprouting directly into the subepicardium, forming a particular bud-like structure (blue arrow in Figure S5B, S5D, and S5E; Videos S7 through S9).

Interestingly, we detected that endocardial sprouting also contributes to the vascularization of the dorsolateral ventricular walls. While this endocardial contribution has been previously suggested,^36^ the later demonstration of nonspecificity of the driver used for such lineage tracing, has been triggering a debate and more recent conflicting lineage tracing studies.^41^ As our 3-dimensional imaging approach (Figure S1B) enabled us to identify the anatomic origin of a given cell/vessel, we observed *Pdgfb*-expressing cells sprouting from the endocardium (Figure 2B). These cells exhibited typical tip cell phenotypes (Figure 2C; Video S10) and coexpression of Fabp4 (Figure 2D), a well-known coronary endothelial marker,^42^ suggesting their eventual acquisition of a coronary endothelial identity. Live imaging on *PdgfbCreERT;R26mTmG* E12.5 heart explants revealed the dynamics of the endocardial angiogenesis. *Pdgfb*-expressing cells emerged from the endocardium, as separate sprouts, which initially did not form part of the growing subepicardial plexus, but acquired an exploratory and migratory tip cell phenotype, to finally make contact with the sprouting front of the subepicardial plexus and incorporate into the coronary plexus (Figure S6; Video S11).

Analysis of *Pdgfb*-expressing endocardial cell distribution revealed that a range of 1% to 3% of all endocardial cells upregulated *Pdgfb* in the ventricles at every timepoint studied, with a peak at E13.5 (Figure S5F) and with no discernible regional differences (Figure S5G). As a result of endocardial angiogenesis, we detected multiple lumenized connections between the endocardium and the developing coronary plexus along both ventricles (Figure 2F; Video S12).

Our findings confirm that the intramyocardial plexus of the ventricular walls, which will give rise to coronary arteries, is formed by sprouting from the SV-derived subepicardial plexus and the endocardium, sprouting in opposite directions. Also, we identify a novel angiogenic wave from the endocardium that contributes to the coronary plexus, and which results in the establishment of lumenized connections between endocardium and the coronary plexus. These connections have been recently referred as endocardial tunnels and are responsible for the rapid arterialization of the postnatal heart.^17^ Our data identified that these endocardial tunnels are already established embryonically as a result of endocardial angiogenic sprouting.

### Prearterial Cells Are Tip Cells Sprouting Toward the Myocardium

**Figure 3.**
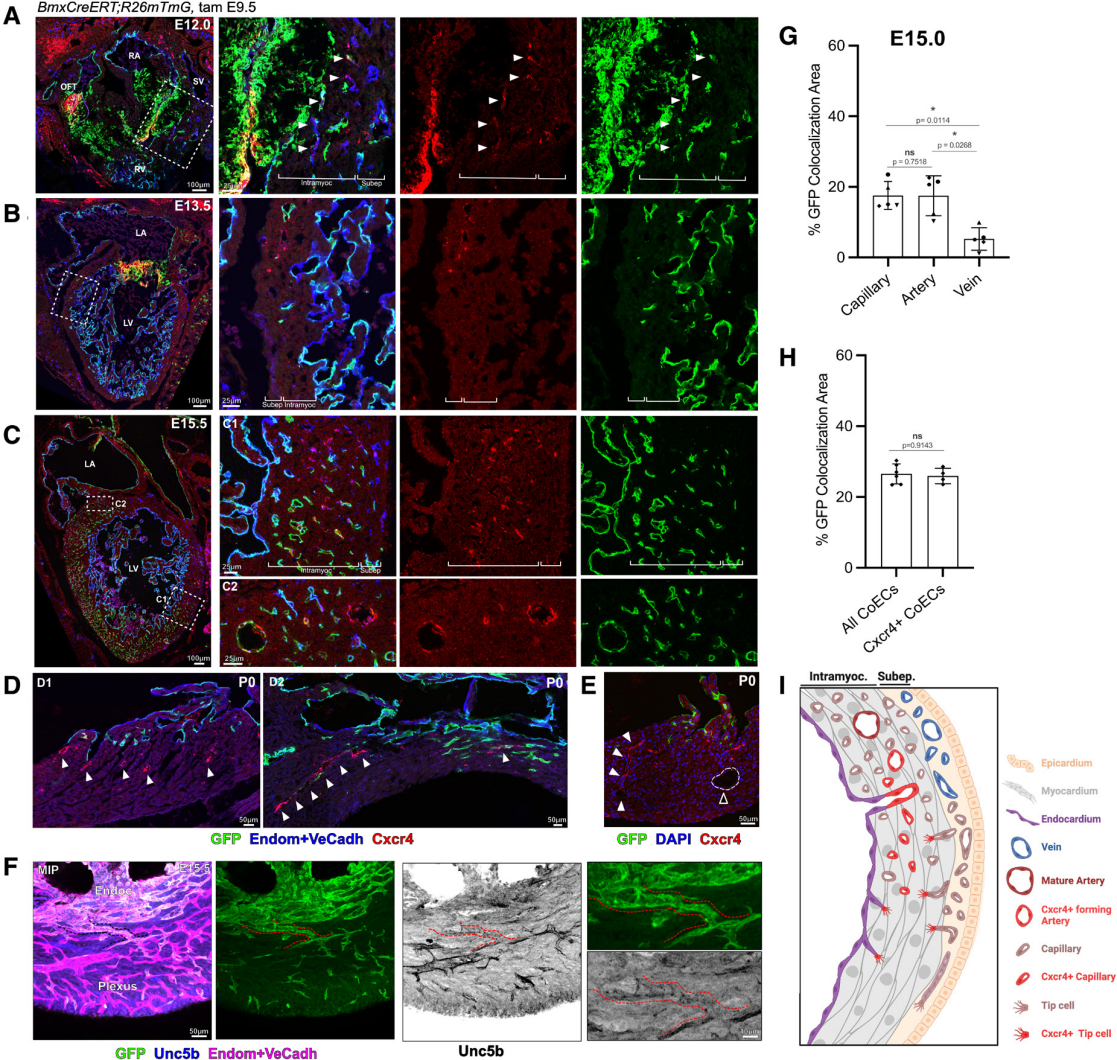
**Expression pattern of the prearterial candidate markers Cxcr4 (C-X-C motif chemokine receptor 4) and Unc5b (Unc-5 netrin receptor B) is restricted to intramyocardial sprouting and forming arteries. A** through **D**, Sections of E12.0—P0 *BmxCreERT;R26mTmG* hearts stained against green fluorescent protein (GFP) (green), Cxcr4 (red), and Endom+VeCadh (blue). **A**, Boxed area shows the atrioventricular region, where the sprouting coronary plexus arises. Subepicardial and intramyocardial coronary endothelial cells (ECs) are well distinguished. While subepicardial ECs do not express Cxcr4, intramyocardial ECs show Cxcr4 expression (arrowheads). A fraction of the intramyocardial ECs is derived from *Bmx*-lineage endocardium. **B**, Boxed area shows the ventricular wall. Again, only intramyocardial ECs express Cxcr4 and GFP. **C**, Boxed areas show 2 regions of the ventricular wall. Cxcr4-expressing ECs are found forming part of the intramyocardial plexus (C1); however, in those areas where remodeling is evident, Cxcr4-expression disappears from the plexus and is only localized in forming arteries (C2). **D.** At P0, Cxcr4-expressing ECs are found in the subendocardial region (D1, white arrowheads), often forming part of vessels that extend from the endocardium (D2, white arrowheads). **E**, Section of P0 *BmxCreERT;R26mTmG* hearts stained against Cxcr4 (red) and DAPI (blue). At this stage, while subendocardial vessels express Cxcr4 (white arrowheads), mature arterial cells do not (black arrowhead). **F**, Maximum intensity projection (MIP) of a segment of the ventricular wall of a E15.0 *BmxCreERT;R26mTmG* heart stained against Unc5b (blue) and Endom+VeCadh (magenta). Endocardium directly connects (dotted line) with intramyocardial vessels that show Unc5b expression. **G**, Quantification of *Bmx*-lineage cells within capillaries, arteries, or veins of the ventricular plexus at E15.0. Quantified in every stack of a 3-dimensional image as percentage of the total area (μm^2^) of *Bmx*-lineage cells (GFP/Endom+VeCadh colocalization) normalized to the area of segmented capillaries, arteries, or veins (Endom+VeCadh) (n=4–7, see dots on bars). *Bmx*-lineage predominantly contribute to capillary and arteries and minimally to veins. Data are mean±SD, every data point (n=5, see paired dots on bars) is an independent individual, *P* values by Friedman test. **H**, Quantification of *Bmx*-lineage contribution to Cxcr4-expressing cell population at E15.0. Quantified in sections as percentage of area (μm^2^) of *Bmx*-lineage cells (GFP/Cxcr4+ colocalization), normalized to total area of CoECs (Endom+VeCadh) or Cxcr4-expressing CoECs (Cxcr4+Endom+VeCadh). Around 25% of intramyocardial Cxcr4-expressing cells are derived from an endocardial *Bmx*-lineage. Data are mean±SD, every data point (n=4–6, see dots on bars) is an independent individual, *P* value by Mann-Whitney *U* test. **I**, Schematic view of a ventricular wall showing the Cxcr4 expression pattern described in **A** through **D**. Cxcr4 is first expressed by tip cells sprouting toward the myocardium, then in intramyocardial capillaries and later it becomes confined in forming arteries. LA indicates left atrium; LV, left ventricle; RA, right atrium; RV, right ventricle; SV, sinus venous; and Tam, tamoxifen.

To further characterize the emergence and distribution of CoEC V/preartery cells, we performed spatiotemporal mapping of Cxcr4 (C-X-C motif chemokine receptor 4) (Figure 3A through 3D) and Unc5b (Unc-5 netrin receptor B) (Figure S7), as they are specific markers of the CoEC V/preartery cluster (Figure 1G; Figure S3B). Additionally, to investigate the origin of this prearterial population, we combined Cxcr4 immunolocalization with the use of a second transgenic mouse line, *BmxCreERT;R26mTmG*^18^ as endocardial lineage tracing tool. We confirmed the specificity of BmxCreERT driver by 3-dimensional analysis upon a single tamoxifen dose at E9.5 (Figure S1A). We validated the specificity of the *BmxCreERT* driver and its labeling efficiency (Figure S8A and S8B; Video S13) and concluded that any green fluorescent protein (GFP)-expressing cell is an endocardial-derivative. Conversely, GFP-negative coronary ECs were assumed to be derived from the SV-derived subepicardial, although the existence of other sources cannot be completely excluded.^43,44^ Additionally, given that *Bmx* is described to be expressed by arterial cells,^18^ we determined that a single-dose tamoxifen administration only drives recombination during 48 hours and, therefore, discarded the occurrence of later recombination events during differentiation of coronary arteries (Figure S9 and S10).

During early phases of coronary vasculature development, Cxcr4 expression was exclusively observed in intramyocardial coronary ECs, when both subepicardial and intramyocardial plexuses were well distinguishable (red in Figure 3A). Given that at this stage, most cells invading the compact myocardium are tip cells, such a pattern is consistent with Cxcr4-expressing tip cells invading the myocardial wall. The mosaicism of GFP labeling in the nascent intramyocardial plexus further illustrates its dual origin from endocardium and SV-derived subepicardial plexus (green in Figure 3A).

As coronary plexus expansion progressed, Cxcr4-expressing capillaries were found extensively within the intramyocardial segment of both ventricular walls (red in Figure 3B and 3C1). In contrast, in areas where remodeling arteries were observed, Cxcr4 became restricted to arterial cells and absent from the surrounding capillaries (Figure 3C2). A similar pattern of expression was exhibited by Unc5b (Figure S7), another previously described tip cell marker^24^ and candidate marker of preartery cells (Figure 1G).

At postnatal stages, Cxcr4-expressing cells were found in the subendocardial zone (Figure 3D) and 3-dimensional analysis confirmed the presence of Unc5b-expressing cells in endocardial tunnels (Figure 3F). As expected,^45,46^ mature arteries lacked Cxcr4 expression (Figure 1G; Figure 3E).

Our analysis of *BmxCreERT2*-induced GFP reporter expression at midgestational stages identified that ≈25% of the total ventricular coronary plexus (Figure 3G, Figure S8C through S8E, Video S14) and ≈25% of the total Cxcr4-expressing prearterial ECs were endocardial-derived (Figure 3H, and green in Figure 3A, 3C and 3E). Since the 3D imaging allowed categorizing the different coronary vessels (Figure S8F; Video S15), our quantification revealed a markedly differential endocardial contribution to arteries, veins and capillaries. Whereas microvascular and arterial vessels comprised of 16% to 20% of endocardial-derived ECs, veins showed only a minor contribution of just 5% (Figure 3G; Figure S8G). This finding reveals a preferential contribution of the endocardium to the prearterial subpopulation and coronary arteries.

### Heterogeneous Intramyocardial and Subepicardial Tip Cell Populations

Given that our identified CoEC V/preartery cells mapped specifically to intramyocardially sprouting tip cells, we investigated whether subepicardial and intramyocardial tip cells exhibit differential transcriptomic signatures and/or regulatory mechanisms.

**Figure 4.**
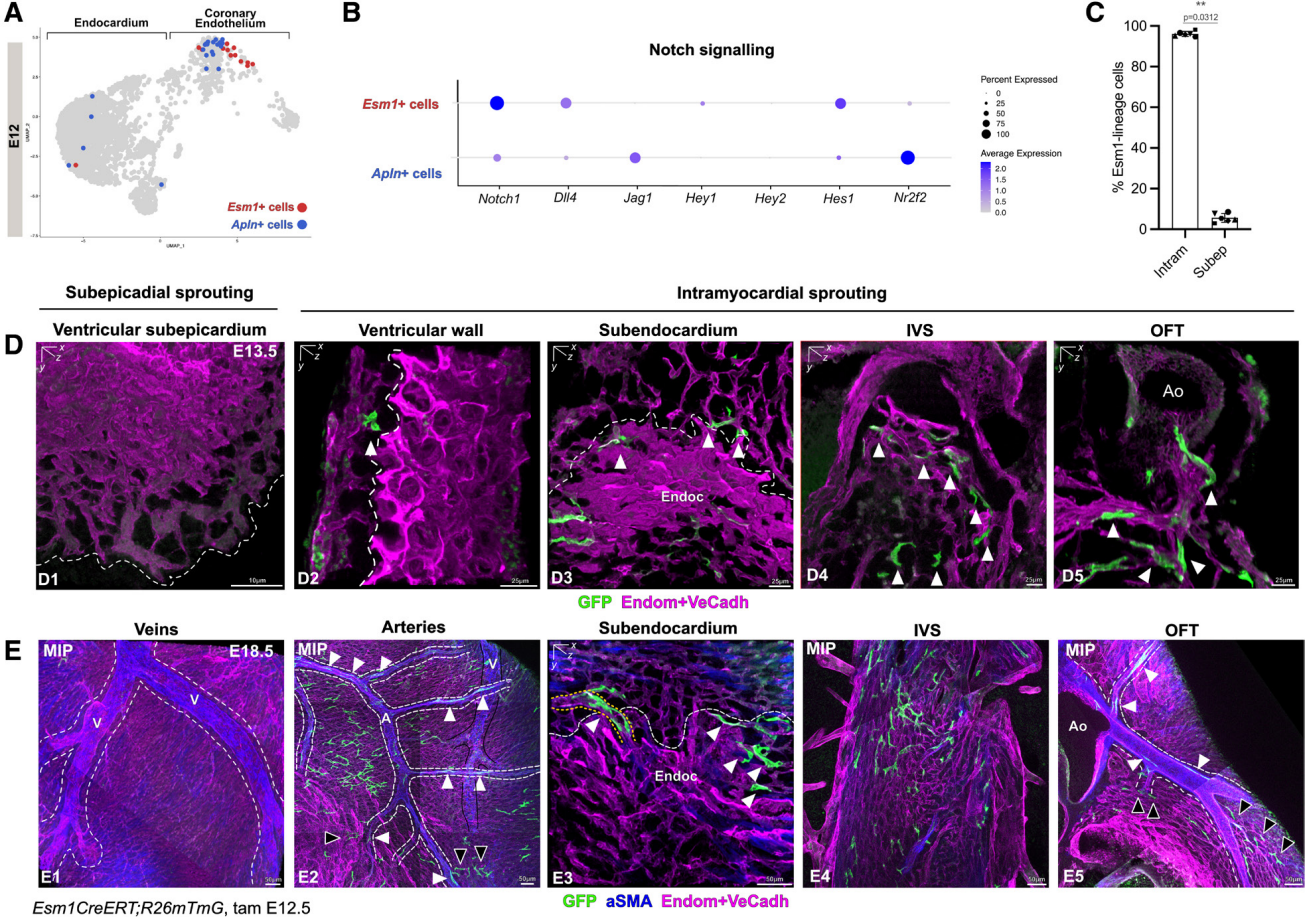
***Esm1*-expressing prearterial cells are specified during intramyocardial sprouting and contribute to arteries. A**, Uniform manifold approximation and projection (UMAP) plot of sorted E12 cardiac endothelial cells (ECs), *Apln*-expressing (blue) and *Esm1*-expressing (red) tip cells are not overlapping. **B**, Dotplot of expression level and frequency of Notch signaling genes among *Esm1*-expressing and *Apln*-expressing tip cells at E12. **C**, Quantification of distribution of *Esm1*-expressing cells within the intramyocardial (**left**) vs the subepicardial (**right**) regions at E13.5. Almost all *Esm1*-expressing cells are found within the intramyocardial region. Data are mean±SD, every data point is an independent individual (n=6, intram vs subep, see paired dots on bars), *P* value by Wilcoxom test. **D**, Three-dimensional rendering of a E13.5 *Esm1CreERT;R26mTmG* heart stained against αSMA (alpha smooth muscle actin) (blue) and Endom+VeCadh (magenta). After 24 hours of tamoxifen induction, *Esm1*-expressing cells are absent from the subepicardial sprouting front (dotted line; D1). *Esm1*-expressing cells (white arrowheads) are observed sprouting toward the myocardium both from the subepicardial plexus (D2) and the endocardium (D3). *Esm1*-expressing cells are also observed sprouting in the interventricular septum (IVS; D4) as well as around the aorta (D5). **F**, Maximal intensity projections (MIPs) of a E18.5 *Esm1CreERT;R26mTmG* heart stained against αSMA (blue) and Endom+VeCadh (magenta). After 6 days of tamoxifen induction, no *Esm1*-lineage cells are detected forming part of veins (white dotted line in E1, black line in E2). *Esm1*-lineage cells are found in arteries (white dotted line in E2) and the surrounding plexus (E2), subendocardial zone (white arrowheads in E3) and vessels that extend from the endocardium (yellow dotted line in E3). *Esm1*-lineage cells are also found in the IVS (E4) as well as in the coronary arteries that stem from the aorta (E5). A indicates artery; intram, intramyocardial plexus; OFT, outflow tract; Subep, subepicardial plexus; Tam, tamoxifen; and V, vein.

*Apelin* (*Apln*) has previously been identified as a subepicardial tip cell marker at E12,^37^ while we identified *Esm1* as a marker of our intramyocardial CoEC V/preartery cell cluster (Figure 1G; Figure S3B). Intriguingly, transcriptomic analysis of the nascent plexus at E12 demonstrated that *Apln*-expressing and *Esm1*-expressing tip cells do not overlap (Figure 4A). Gene set–enrichment analysis of the DEGs between these 2 tip cell subpopulations identified hypoxia and p53 signaling as the top upregulated pathways of *Esm1*-expressing tip cells (Figure S11A). Thus, our data suggest the intriguing existence of 2 distinct tip cell populations that differ in their gene expression profile and differentiation trajectory toward arterial or venous identity. We found no correlation between previously described heterogeneous tip cell populations in the retina^47^ with the cardiac tip cell subpopulations here described (Figure S11B).

Notably, we found *Esm1*-expressing and *Apln*-expressing tip cells exhibited differential expression of key players of the Notch signaling pathway. *Esm1*-expressing cells have higher levels of *Notch1*, *Dll4*, and *Hes1* (Figure 4B). *Apln*-expressing cells have high levels of *Nr2f2*, which has been shown to repress the Notch1 signaling pathway^4^ (Figure 4B).

Previous studies reported that targeted deletion of Dll4 in coronary ECs during embryonic development disrupts prearterial specification and subsequently impairs intramyocardial arterial plexus formation.^16^ Curiously, sprouting from the SV and the formation of the subepicardial venous plexus proceeds almost normally.^16,48^ Despite sprouting itself being reportedly unaffected by Dll4 deletion, a panel of canonical tip cell markers were transcriptionally either up or downregulated in Dll4-deficient cardiac ECs (Figure S11C).^16^ Strikingly, the most significantly downregulated tip cell markers, such as *Esm1*, *Cxcr4*, and *Igfbp3*, corresponded to those which we identified as intramyocardial sprouting markers and related with prearterial specification (Figure S11D). The module score for such downregulated genes in Dll4-deficiency confirmed the enrichment of these markers in our CoEC V/preartery cluster (Figure S11E). Conversely, the module score for the tip cell markers described as upregulated in Dll4-deficient cardiac ECs, such *Apln*, *Adm*, or *Kcne3*, showed an enrichment in our CoEC I/Capillary-to-vein cluster (Figure S11F and S11G), which are thus most likely related to subepicardial sprouting.

Altogether, these findings strongly suggest a highly distinct molecular regulation of intramyocardial and subepicardial tip cells during coronary development.

### *Esm1*-Lineage Tip Cells Contribute to Arteries and endocardial Tunnels

To directly test the hypothesis that *Esm1*-expressing tip cells specifically sprout intramyocardially, we conducted lineage tracing analysis using the *Esm1CreERT* mouse strain,^20^ in combination with the reporter *R26mTmG*.^21^ Reporter recombination was induced with a single dose of tamoxifen at E12.5 and *Esm1*-lineage cells were traced 24 hours (E13.5) and 6 days later (E18.5; Figure S1A).

One day upon reporter labeling induction, almost all *Esm1*-expressing tip cells were located within the myocardium (Figure 4C and 4D), with minimal presence in the subepicardium (Figure 4D1; Video S16). *Esm1*-expressing cells sprouted from both subepicardial plexus and the endocardium at the ventricles (Figure 4D2 and 4D3; Videos S17 and S18) and only from endocardium at the interventricular septum (Figure 4D4; Video S19). Some *Esm1*-expressing cells were also found sprouting around the outflow tract (Figure 4D5; Video S20).

Six days upon reporter labeling induction, all *Esm1*-lineage cells were exclusively located within the intramyocardial plexus and never formed part of subepicardial veins (Figure 4E1). *Esm1*-lineage cells were found in arteries or surrounding capillaries in the ventricular walls (Figure 4E2) as well as in the interventricular septum (Figure 4E4). *Esm1*-lineage cells were frequently found in endocardial tunnels^17^ in the subendocardial zone (Figure 4E3; Video S21), or already integrated in coronary arteries in the proximity of the aorta (Figure 4F5).

Altogether, these data confirm that tip cells, marked by Cxcr4, Unc5b or *Esm1* expression, specifically sprout towards the myocardium and contribute to the formation of coronary arteries, providing evidence for a tip cell-to-artery specification mechanism.

### Prearterial Cells Are Reactivated Upon MI

**Figure 5.**
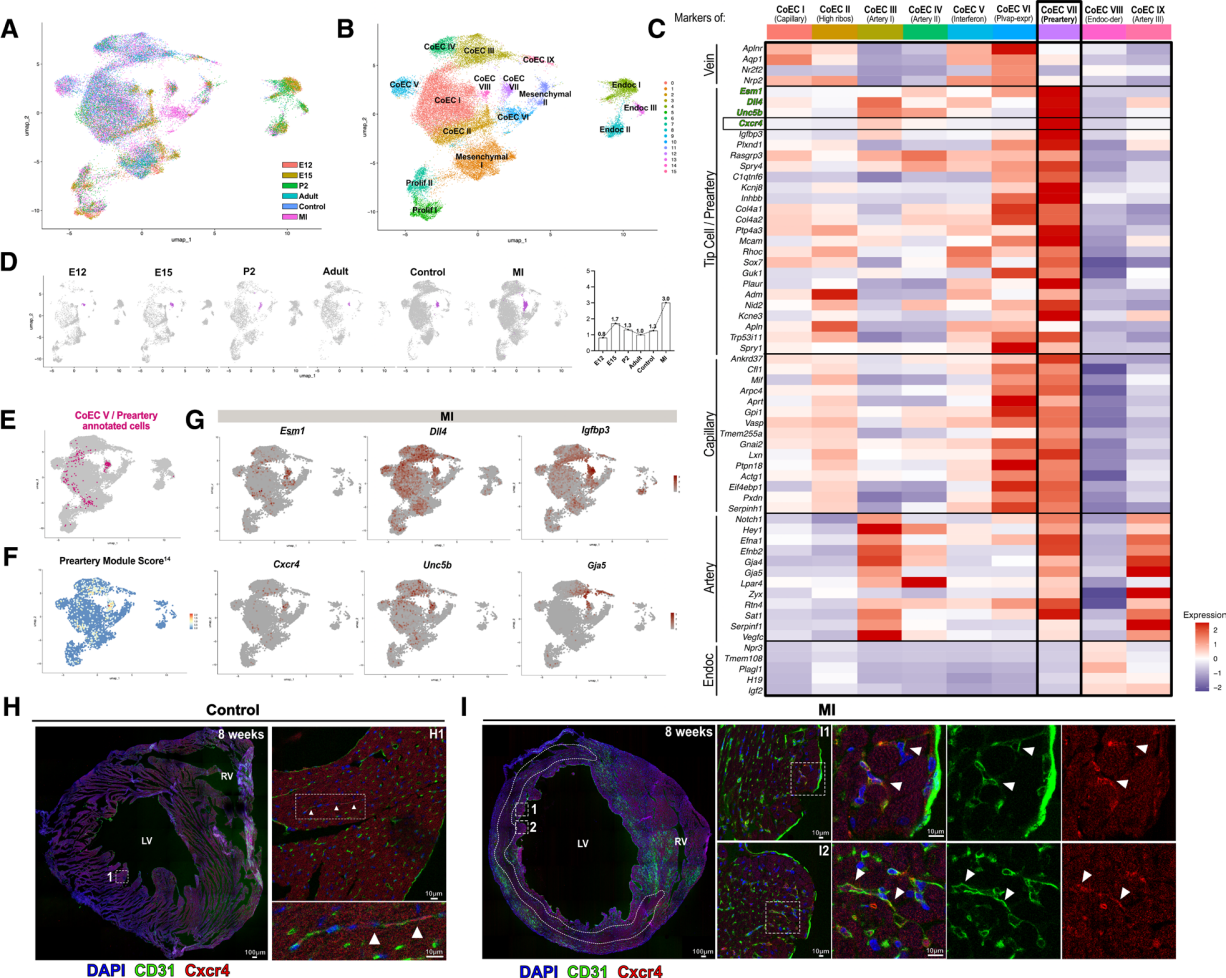
**Prearterial cells increase upon myocardial infarction and are located in the subendocardium. A**, Uniform manifold approximation and projection (UMAP) plot of integrated datasets from sorted cardiac endothelial cells (ECs) from embryonic stages E12 (E12) and E15 (E15), postnatal P2 (P2), 8 weeks adult heart (adult), and 7 dpi myocardial infarction (MI) and corresponding control (control),^49^ color-coded by the data set of origin. **B**, UMAP plot of E12, E15, P2, adult, control, and MI integrated data set. Four groups of clusters are identified, each composed of various clusters: coronary endothelium (CoEC I–CoEC IX), endocardium (Endoc I-Endoc III), mesenchymal endothelium (mesenchymal I and II), and proliferating cells (prolif I and II). **C**, Heatmap of a wide list of known venous, tip cell, capillary, arterial markers, and endocardial markers in all coronary ECs clusters (CoEC I to CoEC IX). **D**, UMAP plot showing CoEC VII/preartery cluster highlighted in purple. Graph shows distribution (%) over time of CoEC VII cluster normalized to total cell number, as also shown in S12C. Preartery cells are detected in all studies stages, with a peak in response to myocardial infarction. **E**, Previously annotated preartery clusters in E12, E15, P2, and adult integrated data set highlighted in pink. These cells mostly map to actual CoEC IX/preartery cluster. **F**, Module score of previously described prearterial marker genes.^14^ CoEC IX/prertery cluster exhibits the highest score for such signature. **G**, Feature marker plots of prearterial markers (*Esm1, Dll4, Igfbp3, Unc5b*, and *Cxcr4*) and arterial marker (*Gja5*) in the myocardial infarction data set. **H**, Section of a 8-week-old wild-type heart stained against CD31 (green) and Cxcr4 (C-X-C motif chemokine receptor 4) (red). Cxcr4-expressing cells are scarce and located in the subendocardium. **I**, Section of a 7-day postinfarction heart stained against CD31 (green) and Cxcr4 (red). Infarcted area is delineated by a dotted line. Cxcr4-expressing cells are more abundant and also located in the subendocardium of the infarcted ventricle. CoEC indicates coronary endothelium; Endoc, endocardium; LV, left ventricle; MI, myocardial infarction; and Prolif, proliferating EC.

We next sought to determine whether the process of tip cell to-artery specification is also active in the response to MI. By integrating publicly available single-cell transcriptomic data from ECs of both control (control) and 7 days postinfarction adult hearts (MI)^49^ with our own data sets spanning developmental stages (E12, E15, P2, and adult), we aimed first, to identify the prearterial signature, and second, to elucidate changes in such cell populations following MI (Figure 5A; Figure S12A). Again, following stringent quality control measures and exclusion of non-ECs, immediate-early gene-expressing ECs, and lymphatic ECs, we conducted unsupervised clustering analysis using uniform manifold approximation and projection, which consistently identified 4 major groups of cardiac endothelial clusters: endocardium, coronary endothelium, proliferating cells, and valvular/mesenchymal (Figure 5B). Annotations of resulting clusters were made based on previous descriptions^49^ and the expression of the top DEGs (Figure 5C; Figure S12B).

Among all the coronary endothelial clusters, we identified a distinct cluster, labeled as CoEC VII (Figure 5D), characterized by enrichment of capillary, tip cell, and arterial markers, consistent with the profile of prearterial cells, including *Esm1*, *Cxcr4*, *Dll4*, *Igfbp3*, *Unc5b*, and *Gja5* (Figure 5C). Notably, tracking unique cell barcodes of previously annotated prearterial cells demonstrated that these cells predominantly mapped to this cluster (Figure 5E) and exhibited a high score for the established prearterial signature^14^ (Figure 5F), solidifying CoEC VII as prearterial cells. Following MI, there was a discernible increase in the abundance of this prearterial cell population (Figure S12C), indicating their potential involvement in the ischemic response. Immunolocalization of Cxcr4, one of the top DEGs identified within the CoEC VII/preartery cluster (Figure 5G), revealed expression in the subendocardial zone in the control (Figure 5H) and, more abundantly, at 7 days postinfarction hearts (Figure 5I).

### Prearterial Cell Transcriptional Signature Is Conserved in Human Embryos

To further explore the conservation of prearterial cells across species, we also analyzed publicly available single-cell transcriptomic data from human cardiac ECs from 13 to 14 weeks of embryonic development.^50^ Employing the same quality control measures and cell filtration as previously described, we conducted UMAP, which once again identified the 4 major groups of cardiac endothelial clusters: endocardium, coronary endothelium, proliferating cells, and valvular/mesenchymal (Figure S13A). Annotations were assigned based on established descriptions^50^ and the expression patterns of the top DEGs (Figure S13F). Remarkably, within the human embryo data set, we identified a distinct population of cells exhibiting enrichment of key markers of sprouting tip cells as well as arterial ECs, closely mirroring the transcriptional signature observed in the murine prearterial cell population (Figure S13C through S13E). This data provide the first evidence for tip cell-to-artery specification in human coronary development.

## DISCUSSION

Tip cell specification is a highly coordinated process that has been extensively studied. It is known to involve multiple signaling pathways, cell-cell interactions, and responses to environmental cues.^51^ Lineage tracing studies in the mouse retina and the zebrafish vasculature showed tip cells are specifically recruited into arteries and excluded from veins,^11–13^ suggesting a close association between tip cell specification and the acquisition of arterial fate. At a regulatory level, the upregulation of Cxcr4 in tip cells, induced by high levels of VEGF and Notch-Rbpj axis activation, was suggested to facilitate the collective migration of tip cells to assemble into arterial vessels.^9,12,13^ Recently, Eph-Ephrin interactions have been proposed to act upstream of Notch and VEGF signaling and play a crucial role in arteriovenous sorting at the sprouting front.^52^ In contrast, in the heart, the specification of prearterial cells was explicitly not associated with sprouting, but defined as individual venous cells within the immature plexus that abruptly upregulate mature artery markers,^14,15^ and later contribute to building coronary arteries. Nr2f2 and Notch pathways counteraction, and their role controlling cell cycle, have been proposed as the master regulators of cardiac prearterial cell specification.^14,16^

Through transcriptional characterization, we demonstrate that cardiac prearterial cells exhibit a unique transcriptional signature, indicative of both capillary and arterial identity, alongside a robust enrichment of tip cell markers, such as *Cxcr4*, *Unc5b*, and *Esm1. Esm1*-lineage tracing confirmed that these tip cells are preferentially incorporated into coronary arteries and endocardial tunnels,^17^ while never found in veins. Overall, we here, for the first time, identify cardiac prearterial cells as sprouting tip cells.

The accumulation of tip cells into arteries has been hypothesized to involve their migration against flow toward developing arteries.^11–13,16^ Similar to the well-documented features of sprouting tip cells, cardiac prearterial cells exhibit a highly migratory and low proliferative profile, which has also been recognized as essential for arterialization.^14,16,31,32^ Our transcriptomic analysis suggests that increased RhoGTPases activity enhances prearterial migration, while p53/Rb signaling, most likely induced by hypoxia,^27^ plays a role in prearterial cell cycle arrest.^11,14,16^ This evidence strongly supports the notion that the acquisition of tip cell features at the sprouting front primes these cells for an arterial fate. Despite prearterial cells expressing mature artery markers before the onset of cardiac blood flow,^14^ flow is widely known to play a critical role in coronary arterialization.^7,9,10^ Further investigation is needed to decipher the role of blood flow in the differentiation of prearterial cells into fully differentiated arteries. Similarly, whether all coronary arterial cells have previously transitioned from tip cells or if additional mechanisms are at play remains an open question.

Our comprehensive 3-dimensional analysis unveils the pattern of angiogenic sprouting, as well as prearterial emergence and distribution during coronary vasculature formation. We identify 2 different advancing angiogenic fronts, 1 progressing superficially within the subepicardium, and the other penetrating the myocardial wall, from both the subepicardial plexus and endocardium. Importantly, we identify a strongly preferential intramyocardial location of Cxcr4- and *Esm1*-expressing tip cells. At initial stages of coronary sprouting, we find no overlapping between *Apln*-expressing and *Esm1*-expressing tip cells, suggesting these 2 subpopulations are mutually exclusive. Up until now, *Apln* and *Esm1* have been considered common canonical tip cell markers.^26^ Differential gene expression analysis between these 2 subpopulations highlights an enrichment of hypoxia-related genes in *Esm1*-expressing intramyocardial tip cells. In contrast to the subepicardium, the intramyocardial region has been shown to be highly hypoxic.^39,53^ Previous studies indeed suggested that subepicardial sprouting from the SV, which will give rise to coronary veins, is a developmentally timed event, while intramyocardial sprouting from the endocardium is associated with a hypoxic environment.^39^

Notably, these subepicardial and intramyocardial tip cell subpopulations exhibit differential Notch signaling, with exclusive Dll4 expression by intramyocardial tip cells. The reexamination of previously described transcriptional changes on the cardiac endothelium upon loss of Dll4^16^ provided further support for the existence of a differential signature between these 2 tip cell populations. Dll4-knockout cardiac endothelium showed downregulation of only a few specific tip cell markers, which we identify as distinctive of the intramyocardial sprouting subpopulation and therefore, of prearterial specification. Consistently, in these mutants, the formation of the intramyocardial plexus and its arterialization is compromised, while the subepicardial venous plexus remains unaffected.^16,48^

Altogether, this evidence suggests that intramyocardial sprouting cells may experience higher hypoxic environment that leads to differential Notch signaling and culminates with differential arteriovenous fate, due to the imprinting of distinct cellular behaviors.

Finally, after confirming that tip cell-to-artery specification plays a role in arterialization during cardiac development, we aimed to elucidate whether this process played a role in response to injury. Neovascularization upon MI has been described to occur by clonal expansion of preexisting ECs in the border zone.^54,55^ Also, the endocardium has been described to contribute to post-MI neovascularization through the formation of the so-called endocardial flowers,^56^ which exhibit many similarities with the recently described endocardial tunnels. Both postnatal endocardial tunnels^17^ and post-MI endocardial flowers^56^ are lumenised connections between the endocardium and the coronary plexus, which undergo progressive arterialization. Curiously, also for both structures, angiogenesis was described to precede arteriogenesis. We here describe that the angiogenic capacity of the endocardium leads to the establishment of endocardial-coronary plexus connections already embryonically. Furthermore, the contribution of *Esm1*-lineage tip cells and their expression of prearterial markers, such as Cxcr4 or Unc5b suggests that arterialization of endocardial-coronary connections progresses through an initial angiogenic sprouting and acquisition of prearterial state. Consistently, *Bmx*-lineage endocardial tracing also determines a preferential contribution of the endocardium to the arterial compartment.

Upon MI, we have detected an increase in Cxcr4-expressing cells in the subendocardium of the infarcted ventricle. This increase aligns with the proportional increase of cells with a prearterial transcriptional signature, characterized by the enrichment of both sprouting, as Cxcr4, and arterial markers, detected in post-MI cardiac samples. This, together with the conserved angiogenic and arteriogenic capacity^17,40,54,56^ of the endocardium suggests that tip cell-to-artery specification from the endocardium might have a role in the ischemic response. Accordingly, the loss of Dll4 in the endocardium results in a drastic reduction of endocardial tunnels and poorer heart function than that seen in infarcted controls.^17^

We propose a revised model of coronary plexus formation and arterial remodeling. Our model suggests first that differential angiogenic programs, originating from various sources, contribute to an integrated coronary plexus. Second, these events determine the subsequent remodeling of the network, by the specification of prearterial cells during intramyocardial sprouting. These cells contribute to the formation of coronary arteries as well as endocardial tunnels that become arterialized. The persistence of prearterial cells into adulthood and the identification of similar transcriptional profiles in different vascular beds of diverse organisms,^11–16,57^ including in human embryos, suggests that prearterial specification may be a conserved mechanism. Notably, we provide evidence for tip cell-to-artery specification to indeed become reactivated upon MI.

Understanding the mechanisms underlying coronary arterialization, and exploring the role of prearterial cells in the adult coronary vasculature as well as in the context of ischemic injury, is key to advancing therapeutic neovascularization strategies. Tailoring such strategies to distinct sprouting mechanisms for the intramyocardial and subepicardial vascular structures may soon become feasible as more insight is gained into the different regulatory and morphogenic principles.

## ARTICLE INFORMATION

### Acknowledgments

The authors thank all members of the Gerhardt laboratory for interesting discussions and comments and especially to Dr Alexandra Klaus-Bergmann and Katja Meier for further technical support. They also thank Marie Altmann and the rest of the Mouse Facility staff at the MDC for excellent animal care, as well as the Advanced Light Microscopy and Image Analysis technology platform, Genomics platform, and Flow Cytometry technology platform at Max-Delbrück Center for Molecular Medicine (MDC). They gratefully acknowledge Prof Ralf Adams for sharing *BmxCreERT* and *Esm1CreERT* mouse strains. Finally, they thank Prof. José María Pérez-Pomares and laboratory members for providing myocardial infarction samples.

### Sources of Funding

This project was supported by the Deutsche Forschungsgemeinschaft (DFG, German Research Foundation, SFB1366—B06 and SFB1470—A04) and by the Deutsches Zentrum für Herz-Kreislauf-Forschung (DZHK; German Center for Cardiovascular Research). E. Cano was also supported by a Postdoctoral Fellowship from Ramon Areces Foundation.

### Disclosures

None.

### Supplemental Material

Supplemental Methods

Figures S1–S13

Tables S1 and S2

Videos S1 and S21

Major Resources Table

References 58–64

## Supplementary Material


