# Supplementary material for: Intramyocardial Sprouting Tip Cells Specify Coronary Arterialization
Source: Circ Res. 2024 Aug 14;135(6):671–84. doi: 10.1161/CIRCRESAHA.124.324868 (PMC11361357; doi:10.1161/CIRCRESAHA.124.324868)
Supplement: Supplementary file 1 [file res-135-671-s001.docx]

**SUPPLEMENTAL MATERIAL**

**Supplemental methods**

**Immunofluorescence staining**

Embryos and pups were fixed in 2% fresh paraformaldehyde (PFA) solution in PBS overnight rocking at 4ºC, washed in PBS, cryoprotected in sucrose solutions, embedded in optimum cutting temperature (OCT) embedding compound (Tissue-Tek), and frozen at -80ºC. Tissue sections were first incubated with blocking buffer (10% donkey serum, 0.1% Triton X-100 and 1% BSA in PBS) for 1 hour at room temperature and then with primary antibodies overnight at 4ºC. The following primary antibodies were used: anti-Cxcr4 (Abcam, ab124824), anti-Endomucin (Santa Cruz, sc-65495), anti-ERα (Santa Cruz, sc-543), anti-Erg (Abcam, ab92513), anti-Fabp4 (Abcam, ab13929), anti-GFP(Abcam, ab13970), anti-ICAM2 (BD Pharmigen 553326), anti-Unc5b (Cell Signaling, 13851), anti-VeCadherin (BD Biosciences, 555289), and anti-αSMA-Cy3 (Sigma, C6198). Alexa fluor-conjugated secondary antibodies (Invitrogen) were incubated for 1-2 hours at room temperature to detect the signals. For some weak signals, HRP-conjugated secondary antibodies and tyramide signal amplification kit (PerkinElmer) were used to magnify the signals. Sections were mount with Vectashield (Vector). All images were captured on a Zeiss LSM-780 confocal microscope. ImageJ software was used as image analysis tool.

**3-dimensional imaging**

Clearing of the dissected embryonic or postnatal hearts were performed with an adapted iDISCO protocol^58^.

Embryonic or postnatal hearts were dissected from the body and fixed in fresh 2% PFA overnight at 4ºC and then thoroughly washed with PBS. Permeabilization was performed with PBT 0.2% for 2h, and then with PBS/0.1% Tween20/0.1% TritonX-100/0.1% Deoxycholate/0.1% NP40/20% DMSO overnight at room temperature. Samples were then blocked with blocking buffer (PBS/0.2%TritonX-100/10%DMSO/0.3Mglycine/6% Donkey) Serum for 12h at room temperature and washed with PBTwH(PBS/0.2% Tween-20 with 10ug/ml heparin) 1h. The incubation of primary antibody was performed in PBTwH / 5% DMSO/ 3% Donkey Serum, shaking for 24h at room temperature. The antibodies used were the same antibodies used for immunofluorescence on sections. The concentration of the primary antibody was 1:25-1:200 depending on the sample size. Afterwards, hearts were washed with PBTwH 1h shaking at room temperature eight times. Alexa Fluor-conjugated secondary antibodies were incubated in PBTwH/3% Donkey Serum overnight shaking at room temperature, at a concentration of 1:50-1:200 depending on the sample size. After thoroughly washing samples with PBTwH again 1h shaking at room temperature eight times. For clearing, samples were dehydrated with increasing concentrations of Tetrahydrofuran solution in H_2_0 (THF): 1h in THF 50%, 1h THF 80% and two times 1h THF 100%, and then in Dicholoromethane until samples sink at the bottom (with a maximum of 5 minutes). Finally, samples were cleared in DiBenzyl Ether (DBE) shaking until the sample was clear. Samples can be stored in DBE at 4ºC until imaged.

For imaging, samples were placed in a drop of DBE on a glass-bottomed dish. Image acquisition was performed with a long working-distance oil-immersion 25x objective on a Zeiss LSM-780 microscope. Z-stacks of a maximum of 460um were acquired. For samples bigger than E12, two different z-stacks acquisition were performed, one from de dorsal side of the heart, and the other from the ventral side.

**3-dimensional images post-processing**

After confocal images acquisition, Zen 3.4 Blue edition software (Zeiss) was used for stitching. Arivis Vision4D software (Zeiss) was used for registering dorsal and ventral Z-stacks when needed. ImageJ was used for manual segmentation. Imaris 9.9.0 (Oxford Instrumentals) was used for 3-dimensional rendering, colocalization analysis and area quantification.

The segmentation of the coronary plexus or the endocardium was performed manually. Navigation through z-stacks of confocal images allowed to determine whether a cell or vessel is in continuation to the subepicardial plexus or, in contrast, is still integrated within the endocardium. The higher intensity of Endomucin staining shown by endocardial cells also served to identify whether a sprouting cell remained connected to the endocardial lining. In those cases of connections of endocardium and coronary plexus, the segmentation boundary was decided arbitrary, by extrapolating the limit between endocardium and coronary plexus of neighbor cells.

**Live imaging on explanted hearts**

Live-imaging on explanted embryonic heart was performed as previously described^43^. E12.5 *PdgfbCreERT;R26mTmG* embryos were dissected in PBS supplemented with 10%FBs, penicillin (100 U/ml) and streptomycin (100ug/ml). Hearts were isolated and transferred to Hepes-buffered DMEM/F12 medium supplemented with 2% FBS and penicillin-streptomycin. Hearts then were embedded into a 0.5% low melting point agarose gel (Sigma-Aldrich) in a glass-bottomed culture dish (MatTek) and bathed in the same medium used for heart dissection. Images were captured in a 3i spinning-disc confocal microscope every 10 min for 15 hours. During image capture the culture chamber was maintained at 37 °C in a 5% CO2 humidified atmosphere.

**Single cell suspension preparation and cell sorting**

Single-cell suspension of ECs was prepared as described previously^59^. C57Bl6 8 weeks old adult animals, P2 postnatal, E15 and E12 were used for single-cell suspension preparation. Adult animals were sacrificed by cervical dislocation while embryonic and postnatal animals were decapitated. Adult and postnatal hearts were chopped with a scalpel until getting 1 to 2mm pieces of tissue. The enzymatic digestion was adapted to every stage analyzed: for E12, 20min of Collagenase/Dispase (Sigma-Aldrich) (1mg/ml), for E15, 30min of Collagenase/Dispase (1mg/ml), and for P2 and adult hearts, 20 min of Collagenase II (500units/ml) (Worthington) plus a second digestion step with Collagenase/Dispase for 30min. All steps were performed at 37ºC, under agitation and pippeting up and down the solution within the tubes every 5min. To stop the enzymatic reaction 10ml of cold FAC buffer (HBSS/2%FBS/10mM Hepes) was added to the samples and then spinned down (400G, 5 min). For adult samples, red blood cells lysis step (Invitrogen) was performed. After enzymatic digestion, cell debris removal solution (Miltenyi) was performed and finally, all suspension filtered through a 40um strainer.

Single-cell suspensions were incubated with primary antibodies in FACS buffer for 20 min on ice. The following antibodies were used: APC-conjugated anti-CD31 (Thermo Fisher, 17-0311-80) and PE-conjugated anti-CD45 (BD Biosciences, 553081). Samples were centrifuged (400G, 5 min) and resuspended on FACS buffer and stored on ice until applied to the flow cytometer. CD31+/CD45- cells were sorted on an Aria II into 1.5ml tubes with EBM2 media. Compensation controls were set up for each single channel. After completion of sorting, 10% DMSO was added and cell suspension frozen at -80ºC. Several rounds of single-cell suspension preparation, sorting and freezing was repeated for embryonic samples in order to get a significant number of cells: 8 weeks old adult animals (n=6), P2 postnatal (n=21), E15 (n=48) and E12 (n=37).

Frozen vials of sorted CD31+/CD45- cells from E12, E15, P2 and 8wo adult hearts were thawed in a water bath at 37ºC. For E12 and E15, a dead cell removal kit (Miltenyi) was applied. For P2 and adult, re-sorting by flow cytometry and selection of live cells was performed.

**Library preparation and 10X Chromium sequencing**

Cell suspension was adjusted to 400 to 1000 cells per microliter and loaded on the Chromium controller (10X Genomics) with a targeted cell recovery of 8000 to 10000 per reaction. 3′ gene expression libraries were prepared according to the manufacturer’s instructions of the v2 Chromium Single Cell Reagent Kits (10X Genomics). Quality control of cDNA and final libraries was done using Bioanalyzer 2100 (Agilent). Libraries were sequenced using HiSeq 4000 (Illumina) targeting a depth of 25000 reads per cell.

**Transcriptome mapping**

After sequencing, the sequence data were mapped to the mouse reference genome (mm10 prebuild references v 2.1.0) provided by 10X Genomics using the CellRanger suite (v.2.2). The count matrices generated by CellRanger as followings were used for further analysis. Mapping quality was assessed using the CellRanger summary statistics.

**Empty droplets removal and doublet estimation**

Empty droplets were identified by Emptydrops, which is implemented in the CellRanger workflow. After removal of empty droplets, we applied scrublet per sample^60^ to assign a doublet score (scrublet_score) to the metadata container of each cell.

**Cell quality control and filtering**

Downstream analysis employed the concatenated filtered feature-barcode matrices, using Seurat^61^. Genes were filtered out when they were expressed in less than three cells. The filtering cut-off was decided based on the distribution of data quality and the estimated doublet ratio provided by 10X Genomics. We applied individual cell filtering criterion: cells from E12 were filtered for counts (nCount_RNA <= 4000), genes (nFeature_RNA <= 1500), mitochondrial genes (percent.mt <= 10%), and scrublet score (scrublet_score <= 0.15). Cells from E15 were filtered for counts (nCount_RNA <= 3000), genes (nFeature_RNA <= 1300), mitochondrial genes (percent.mt <= 10%), and scrublet score (scrublet_score <= 0.15). Cells from P2 were filtered for counts (nCount_RNA <= 12000), genes (nFeature_RNA <= 4000), mitochondrial genes (percent.mt <= 20%), and scrublet score (scrublet_score <= 0.15). Cells from Adult were filtered for counts (nCount_RNA <= 5000), genes (nFeature_RNA <= 2000), mitochondrial genes (percent.mt <= 20%), and scrublet score (scrublet_score <= 0.15).

**Cell cycle score calculation**

Cell cycle score were calculated using CellCycleScoring function integrated in Seurat. Previously reported cell cycle genes^62^ are used for the reference of cell cycle genes after converting the human gene list into mouse orthologues using biomaRt (v2.52.0).

**Dimensionality reduction, clustering, and analysis of differentially expressed genes**

After read count normalization and log2-transformation, top 2000 highly variable genes were selected. Then, regression of UMI counts, percent mitochondrial gene expression and cell cycle gene expression was performed. Prior to manifold construction using UMAP, top 30 principal components were harmonized by anchored canonical correlation analysis (CCA)^63^. Cells were clustered using the original Louvain algorithms^64^. For all datasets, nonendothelial subtypes (e.g. blood and immune cells, cardiomyocytes, smooth muscle, fibroblasts) as well as a small number of lymphatic cells were removed. Likewise, immediate early genes-expressing cells were also removed as considered stressed cells during the single-cell suspension preparation process.

DEGs of each cluster were calculated using FindAllMarkers function of Seurat with the Wilcoxon rank sum test. For the marker gene computation, we selected genes expressed in at least 25% of cells in either of the populations and with a log2-transformed fold change of at least 0.25. Genes with adjusted P value < 0.05 were called as DEGs.

**Left anterior descending coronary artery ligation**

Myocardial infarction (MI) was induced in mice through the ligation of the left anterior descending (LAD) coronary artery on 8 to 12 weeks C57Bl6/J mice. Prior to surgery, mice were anesthetized using vaporized isoflurane, intubated with a 20G intravenous catheter, and placed on a mechanical ventilator for respiratory support. Additionally, to prevent hypothermia, animals were positioned on a heating pad to maintain body temperature throughout the procedure. The surgical site was prepared by performing a left thoracotomy at the fourth or fifth intercostal space, allowing access to the heart. Subsequently, muscles overlying the chest cavity were dissected to expose the LAD coronary artery. Using a 7/0 nonabsorbable ethylene suture, the LAD artery was permanently ligated, ensuring complete occlusion. Verification of successful ligation was confirmed visually by observing anemia and akinesis of the apex and anterolateral wall. Following the confirmation of coronary occlusion, the thoracic cavity was closed in layers to minimize the risk of postoperative complications. Upon completion of the surgery, mice were extubated and monitored closely until fully recovered. To facilitate recovery, mice were kept warm and provided with appropriate postoperative care. Both healthy and 3 days postinfarcted mice were sacrificed and subjected to fixation and subsequent immunostaining.

**Integration of single-cell transcriptomic available data from control and 7dpi cardiac endothelium**

Publicly available datasets were obtained from the Gene Expression Omnibus repository GSE132880^49^. Single-cell RNA seq was performed on FACs sorted CD31+ Confetti+ Podoplanin- cell populations from four control and four 7dpi adult mouse hearts from *PdgfbCreERT2;Rosa26Brainbow2.1*.

Downstream analysis employed the concatenated filtered feature-barcode matrices, using Seurat^62^ . Cells with >0.01 and >0.95 percentiles of nCount and nFeature and >10% mitochondrial genes (percent.mt) were filtered out. Following data filtering, datasets (E12, E15, P2, Adult, Control and 7dpi) were merged to create a unified dataset for downstream analysis. The merged dataset underwent SCT normalization, top 2000 highly variable genes selected and regression of percent mitochondrial gene expression was performed. Integration of datasets was performed using Harmony. Prior to manifold construction using UMAP, top 40 principal components were harmonized by anchored canonical correlation analysis (CCA)^59^. Cells were clustered using the original Louvain algorithms^60^. Following already described criteria, non-endothelial subtypes (e.g. blood and immune cells, cardiomyocytes, smooth muscle, fibroblasts, lymphatic cells and immediate early genes-expressing cells were removed. DEGs of each cluster were calculated using FindAllMarkers function of Seurat with the Wilcoxon rank sum test. For the marker gene computation, we selected genes expressed in at least 25% of cells in either of the populations and with a log2-transformed fold change of at least 0.25. Genes with adjusted P value < 0.05 were called as DEGs.

**Analysis of single-cell transcriptomic available data of human cardiac embryonic endothelium**

Publicly available datasets were obtained from the Gene Expression Omnibus repository GSE195911^50^. Single-cell RNA seq was performed on FACs sorted cardiac ECs isolated by FACS from ventricular tissue obtained from two human fetuses at 13 and 14weeks of gestation.

Downstream analysis employed the concatenated filtered feature-barcode matrices, using Seurat^61^ . Cells with >0.01 and >0.95 percentiles of nCount and nFeature and >10% mitochondrial genes (percent.mt) were filtered out. Following data filtering, datasets (13 and 14 weeks) were merged to create a unified dataset for downstream analysis. The merged dataset underwent SCT normalization, top 2000 highly variable genes selected and regression of percent mitochondrial gene expression was performed. Integration of datasets was performed using Harmony. Prior to manifold construction using UMAP, top 40 principal components were harmonized by anchored canonical correlation analysis (CCA)^59^. Cells were clustered using the original Louvain algorithms^60^. Following already described criteria, non-endothelial subtypes (e.g. blood and immune cells, cardiomyocytes, smooth muscle, fibroblasts, lymphatic cells and immediate early genes-expressing cells) were removed. DEGs of each cluster were calculated using FindAllMarkers function of Seurat with the Wilcoxon rank sum test. For the marker gene computation, we selected genes expressed in at least 25% of cells in either of the populations and with a log2-transformed fold change of at least 0.25. Genes with adjusted P value < 0.05 were called as DEGs.

**Supplemental figures**

**
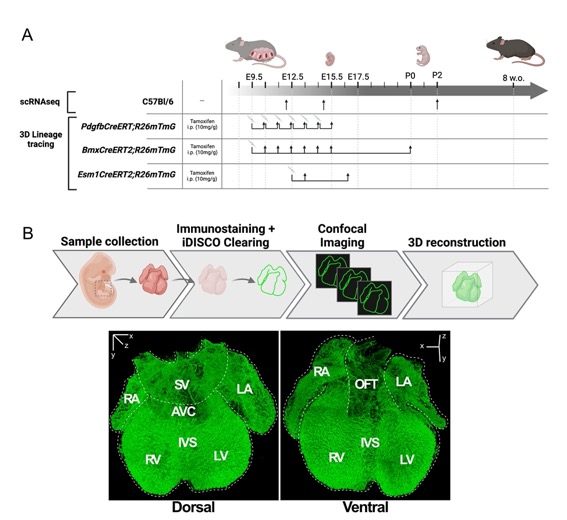
**

**Figure S1. Methods summary**

**A.** scRNAseq and lineage-tracing strategy. Mouse strains, tamoxifen administration and analysis timepoints (arrows).

**B.** 3-dimensional imaging pipeline.

AVC indicates atrioventricular canal; IVS, interventricular septum; LA, left atrium; LV, left ventricle; OFT, outflow tract; RA, right atrium; RV, right ventricle; SV, sinus venous.


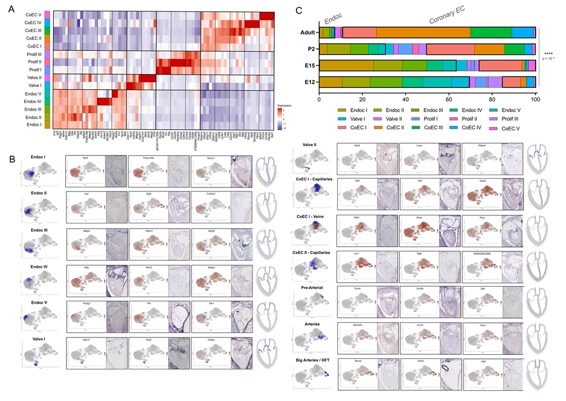


**Figure S2. Validation of annotated clusters by *in situ hybridization* of top DEGs**

**A.** Heatmap of top 5 marker genes of every annotated cluster**.**

**B.** UMAP plot with every cluster highlighted in blue (left), panel with feature marker plots of three top marker genes of each cluster and corresponding ISH images from E14.5 wildtype embryos (middle) and schematic of the observed signal in the ISH images (right). ISH images are from GenePaint database^18^.

**C.** Percentage is calculated by cell number of every cluster, normalized to total cell number, P value by χ2 test.

**
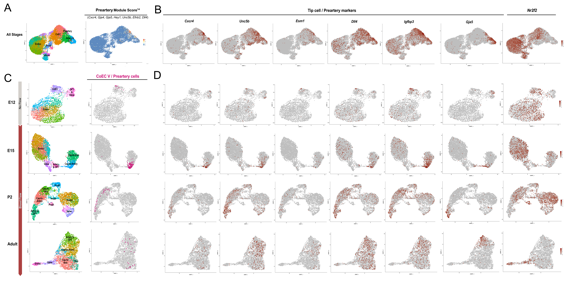
**

**Figure S3. Tip cell signature of prearterial cells is stable throughout life.**

**A.** UMAP of E12, E15, P2 and Adult integrated dataset and module score of previously described prearterial marker genes^14^.

**B.** Feature marker plot of prearterial and tip cell markers (*Cxcr4, Unc5b, Esm1, Dll4, Igfbp3*), arterial marker (*Gja5*) and venous marker (*Nr2f2*) in the UMAP of the integrated datasets.

**C.** UMAP of individual datasets (first column) and mapping of prearterial cells annotated as CoEC V/ Preartery in the integrated UMAP (second column, compare with fig. 1F).

**D.** Feature marker plot of prearterial and tip cell markers (*Cxcr4, Unc5b, Esm1, Dll4, Igfbp3*), arterial marker (*Gja5*) and venous marker (*Nr2f2*) in the UMAP of the individual datasets.

**
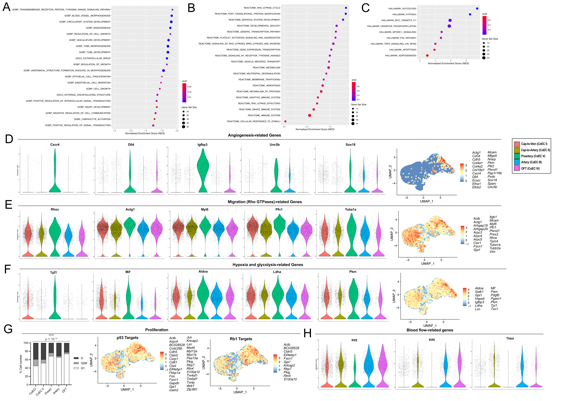
**

**Figure S4. Prearterial cells show an angiogenic, migratory, glycolytic and low proliferative transcriptional profile.**

**A-C.** Gene set enrichment analysis of CoEC V/Preartery cluster in comparison with the rest of CoEC clusters (CoEC I to CoEC IV) for GO-terms (A), reactome (B) and hallmarks (C).

**D.** Violin plots of angiogenesis-related genes in all CoEC clusters and module score of all identified enriched genes of the corresponding GSEA. CoEC V/Preartery cluster shows increased levels in comparison with other CoEC clusters.

**E.** Violin plots of Rho GTPases-related genes in all CoEC clusters and corresponding module score on the integrated UMAP. CoEC V/Preartery cluster shows increased levels in comparison with other CoEC clusters.

**F.** Violin plots of hypoxia-related and glycolysis-related genes in all CoEC clusters and corresponding module score on the integrated UMAP. CoEC V/Preartery cluster shows increased levels in comparison with other CoEC clusters.

**G.** Percentage of cells that are in G1, G2M and S cell cycle phase in each CoEC cluster. CoEC V/Preartery cluster proliferation levels are reduced when compared with capillary cells and show similar levels to mature arteries (left). Percentage is calculated by number of cells categorized by cell cycle score algorithm (see methods), normalized to total cell number for every cluster of interest, P value by χ2 test. Module score on the integrated UMAP of p53 and Rb1 targets found to be enriched in CoEC V/Preartery cluster in comparison with the rest of the CoEC clusters.

**H.** Violin plots of blood flow-induced genes in all CoEC clusters. CoEC V/Preartery cluster shows reduced levels in comparison with other CoEC clusters.

**Fig
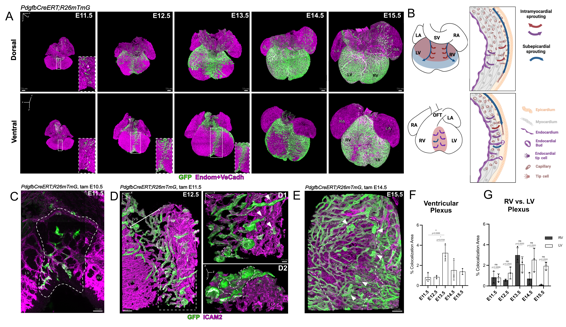
Figure S5. Distinct developmental sources with opposing sprouting directions vascularize the myocardial walls.**

**A.** 3-dimensional rendering of E11.5-E15.5 *PdgfbCreERT;R26mTmG* hearts stained against Endom+VeCadh (magenta). Upper row shows dorsal view, lower row shows ventral view.

**B.** Sprouting directions and sources of coronary plexus formation in dorsal and ventral regions of the heart. At the dorsal side (upper row), the subepicardial plexus, which originates at the SV, sprouts radially within the subepicardium to expand the plexus (blue arrow), and tangentially towards the underlying myocardium to form the intramyocardial plexus /red arrow). Simultaneously, endocardial cells sprout from the opposite direction to also vascularize the myocardium (purple arrow). At the ventral side (lower row), multiple individual endocardial cells sprout towards the myocardium. Few endocardial cells that sprout directly into the subepicardium form bud-like structures. Both dorsal and ventral plexus anastomose to close the network.

**C.** 3-dimensional rendering of the IVS region (dotted line) of a E11.5 *PdgfbCreERT;R26mTmG* heart stained against ICAM2 (magenta). *Pdgfb*-expressing tip cells (green) are sprouting from the endocardium (magenta).

**D.** 3-dimensional rendering of the IVS region of a E12.5 *PdgfbCreERT;R26mTmG* hearts stained against ICAM2 (magenta). D1 boxed area shows numerous *Pdgfb*-expressing endocardial sprouts (white arrowheads), some of these sprouts form bud-like structures (dotted line). D2 shows the same boxed area but 90º rotated in x axis. Endocardial buds (dotted line) are sprouting directly to the subepicardium.

**E.** 3-dimensional rendering of the ventral side of a RV from a E15.5 *PdgfbCreERT;R26mTmG* hearts stained against ICAM2 (magenta). Subepicardial plexus is progressing (dotted line) and anastomoses with the endocardial-derived intramyocardial plexus. Endocardial buds are often observed (white arrowheads).

**F.** Quantification of endocardium expressing *Pdgfb* in the ventricles along development. Quantified in every stack of a 3-dimensional image as percentage of the area (μm^2^) of *Pdgfb*-expressing endocardial cells (GFP/Endom+VeCadh colocalization), normalized to the area of segmented endocardium (Endom+VeCadh) of the ventricles. Data are mean ± SD, every data point (n=3, see dots on bars) is an independent individual. Non-parametric Kruskal-Wallis test was performed, only significant P values are shown in figure (non-significant P values are: E11.5 vs E12.5=0.8908, E11.5 vs. E14.5=0.2724, E11.5 vs. E15.5=0.1200, E12.5 vs. E14.5=0.3369, E12.5 vs. E15.5=0.1563, E13.5 vs. E14.5=0.1095, E13.5 vs. E15.5=0.2529, E14.5 vs. E15.5=0.6474).

**G.** Quantification of endocardium expressing *Pdgfb* in RV versus LV along development. Quantified as in F. Data are mean ± SD, every data point is an independent individual (n=3, LV vs. RV, see paired dots on bars), P value by Wilcoxom rank-sum test.

Intramyoc indicates intramyocardial; IVS, interventricular septum; LA, left atrium; LV, left ventricle; OFT, outflow tract; RA, right atrium; RV, right ventricle; Subepic, subepicardial; Tam, tamoxifen.

**
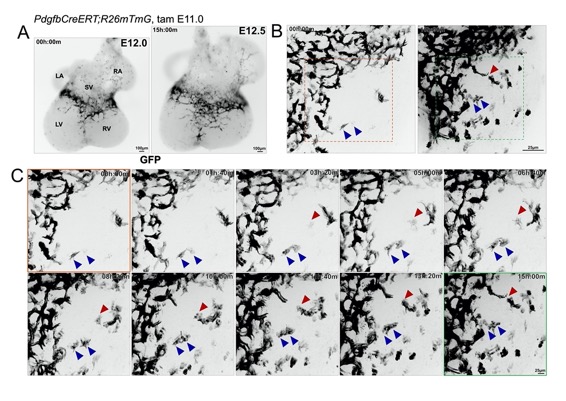
**

**Figure S6. Endocardial tip cells show an exploratory phenotype and make contact with subepicardial plexus.**

**A.** Dorsal view of a E12.0 *PdgfbCreERT;R26mTmG* explanted heart before (t=0) and after (t=15h) live-imaging. The dorsal coronary plexus (GFP, black) progression is comparable to the *in vivo* situation.

**B.** Magnification of RV sprouting front of a E12.0 *PdgfbCreERT;R26mTmG* explanted heart before (t=0) and after (t=15h) live-imaging.

**C.** Time-lapse of SV-derived plexus sprouting front over the RV. *Pdgfb*-expressing endocardial cells (red and blue arrowheads) appear actively attracted by the SV-derived plexus and finally make contact with its sprouting front.

LA indicates left atrium; LV, left ventricle; RA, right atrium; RV ,right ventricle; SV, sinus venosus; Tam, tamoxifen.


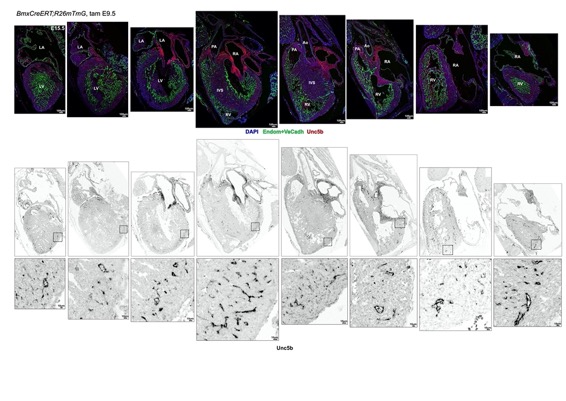


**Figure S7. Unc5b-expressing prearterial cells localize in the intramyocardial segment.**

**A.** Left-to-right serial sections of a E15.5 *BmxCreERT;R26mTmG* heart, stained against Unc5b (red), Endom+VeCadh (green) and DAPI (blue). In the bottom panel, Unc5b expression is showed in gray scale for better visualization. Boxed areas show a magnification of panel above. Unc5b is expressed by the intramyocardial plexus and by vessels that showed signs of arterialization.

Ao indicates oorta; IVS, interventricular septum; LA, left atrium; LV, left ventricle; PA, pulmonary artery; RA, right atrium; RV, right ventricle; Tam, tamoxifen.


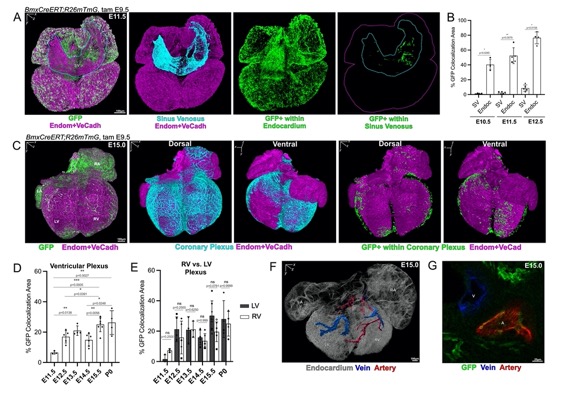


**Figure S8. *BmxCreERT;R26mTmG* is a suitable line for endocardial tracing.**

**A.** 3-dimensional rendering of a E11.5 *BmxCreERT;R26mTmG* heart. SV is shown in cyan, while *Bmx*-lineage cells within the SV or endocardium are shown in green. *Bmx*-lineage cells found within the SV are located close to the transition area where SV drains to RA. Single dose tamoxifen at E9.5 leads to 80% of all endocardial cells labeled at E12.5 while only 8% of SV ECs.

**B.** Quantification of *Bmx*-lineage cells within SV or endocardium within the tamoxifen recombination window (E9.5-E12.5). Quantified as percentage of area (μm^2^) of *Bmx*-lineage cells (GFP/Endom+VeCadh colocalization), normalized to total area of corresponding segmented SV or endocardium (Endom+VeCadh). *BmxCreERT* drives labelling predominantly in the endocardium whereas minimally in the SV. Data are mean ± SD, every data point (n=4-5, see dots on bars) is an independent individual, P value by Mann-Whitney U test.

**C.** 3-dimensional rendering of a E15.0 *BmxCreERT;R26mTmG* heart. *Bmx*-lineage derived coronary ECs (green) distribute broadly within the coronary plexus (cyan) of the ventricles.

**D.** Quantification of *Bmx*-lineage cells within the ventricular plexus along development. Quantified in every stack of a 3-dimensional image as percentage of the area (μm^2^) of *Bmx*-lineage cells (GFP/Endom+VeCadh colocalization), normalized to the area of the coronary plexus of the ventricles (Endom+VeCadh). Data are mean ± SD, every data point (n=4-5, see dots on bars) is an independent individual. Non-parametric Kruskal-Wallis test was performed, only significant P values are shown in figure (non-significant P values are: E11.5 vs E12.5=0.1077, E11.5 vs. E14.5=0.2338, E12.5 vs. E13.5=0.2935, E12.5 vs. E14.5=0.5826, E12.5 vs. P0=0.0962, E13.5 vs. E14.5=0.1079, E13.5 vs. E15.5=0.4218, E13.5 vs. P0=0.5609, E15.5 VS. P0=0.8829).

**E.** Quantification of *Bmx*-lineage cells within the plexus of LV and RV along development. Quantified in every stack of a 3-dimensional image as percentage of the area (μm^2^) of *Bmx*-lineage cells (GFP/Endom+VeCadh colocalization), normalized to the area of the coronary plexus of LV and RV (Endom+VeCadh). Data are mean ± SD, every data point is an independent individual (n=3-7, LV vs. RV, see paired dots on bars), P value by Wilcoxom rank-sum test.

**F**. Post-processing segmentation of developing coronary arteries (red) and veins (blue) in a *BmxCreERT;R26mTmG* E15.5 heart. Segmentation is based in their connection to the RA through the coronary sinus (veins) or to the aorta (arteries).

**G.** Single z plane of heart shown in F*. Bmx*-lineage cells (green) are preferentially located integrated in coronary arteries (red) rather than coronary veins (blue).

A indicates artery; Endoc, endocardium; LA, left atrium; LV, left ventricle; RA, right atrium; RV, right ventricle; SV, sinus venosus; V, vein.


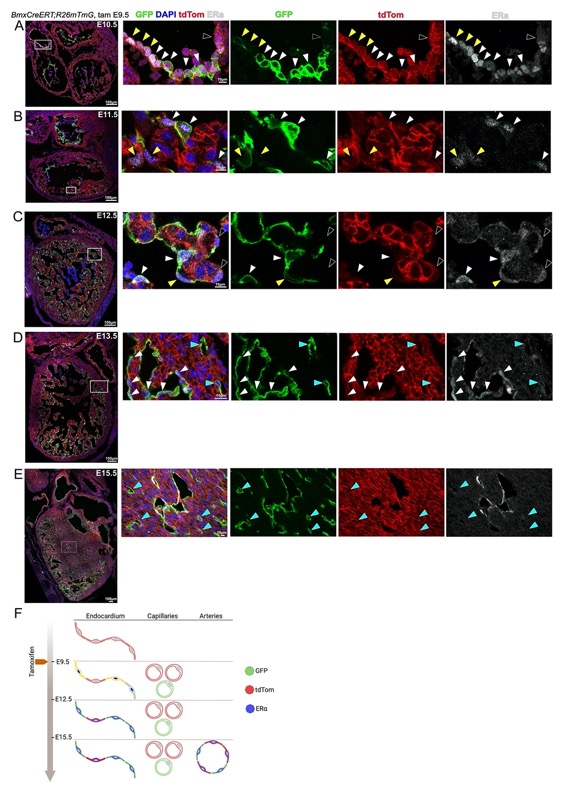


**Figure S9. *BmxCreERT* mediated recombination only occurs during a 48h time-window upon a single E9.5 tamoxifen injection.**

**A-E**. Sections of E11.5-E15.5 *BmxCreERT;R26mTmG* hearts, stained against Estrogen Receptor α (ERα) to analyze the distribution of the CreERT (expressed from the *Bmx* promoter) and tdTomato (tdTom) proteins along development. Black arrowheads point to GFP-negative/tdTom-positive cells; yellow arrowheads to GFP-positive/tdTom-positive cells; white arrowheads to GFP-positive/tdTom-negative/ERα-positive cells; and cyan arrowheads to GFP-positive/tdTom-negative/ERα-negative cells.

**F.** Schematic representation of GFP, tdTom and ERα signals detected in panels above.

By analyzing the colocalization of GFP and tdTom we observe recombination exclusively in endocardial cells and only during the first 48h upon tamoxifen administration. Also, in this time window, ERα localizes to the nuclei of *Bmx*-expressing endocardial cells. From E12.5 onwards, ERα is found in the cytoplasm of all *Bmx*-expressing endocardial cells. In capillaries, we never observe CreERT or tdTom expression, indicating that GFP labeling is evidence for being derived from the *Bmx*-lineage endocardium.

In arteries, we detect ERα expression, which recapitulates *Bmx* expression. However, ERα is always located at the cytoplasm, so the GFP labeling in arterial ECs is not due to autonomous recombination in arteries, but a consequence of earlier lineage recombination (as endocardial cells). Also, we observe ERα-expressing arterial cells that lack GFP, providing further evidence that the earlier tamoxifen injection is no-longer leading to any recombination event at this later stage.

Tam indicates tamoxifen.

**
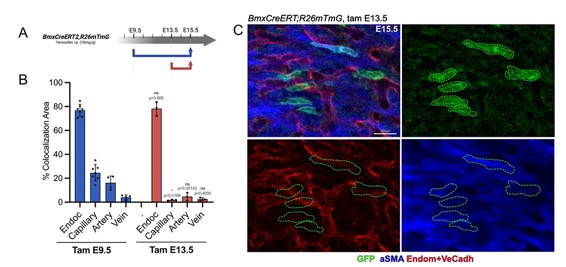
**

**Figure S10. *Bmx* is not upregulated by ECs within the *BmxCreERT* recombination window upon a single E9.5 tamoxifen injection.**

**A.** Lineage-tracing strategy. Comparison of E15.5 *BmxCreERT;R26mTmG* hearts when tamoxifen is administered at different timepoints (E9.5 and E13.5).

**B.** Percentage of *Bmx*-lineage cells within endocardium or coronary plexus, segmented in capillaries, arteries or veins at E15.5. Quantified in every stack of a 3-dimensional image as GFP/Endom+VeCadh colocalization area (μm^2^), normalized to the area of segmented capillaries, arteries or veins. E13.5 tamoxifen administration leads to a decreased labeling of *Bmx*-lineage derived coronary plexus. Data are mean ± SD, every data point (n=3-7, see dots on bars) is an independent individual, P value by Mann-Whitney U test.

**C.** Section of a E15.5 *BmxCreERT;R26mTmG* heart, stained against αSMA and Endom+VeCadh. Only when tamoxifen is administered at E13.5, *Bmx*-lineage non-endothelial cells are observed (green outline), confirming that E9.5 single-dose tamoxifen does not lead to any recombination 72 hours later (at E13.5).

Endoc indicates endocardium; Tam, tamoxifen.


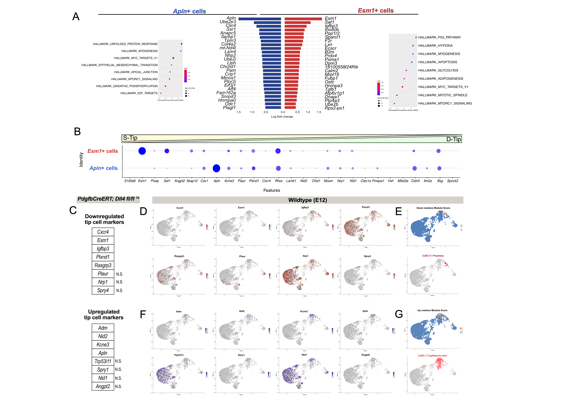


**Figure S11. Differential transcriptional signature of *Esm1*-expressing and *Apln-*expressing tip cell subpopulation.**

**A.** DEGs between *Apln*-expressing and *Esm1*-expressing tip cells and corresponding gene set enrichment analysis for hallmarks of each population at E12. *Esm1*-expressing tip cells show enrichment of p53 and hypoxia pathways genes.

**B.** Dotplot of expression level and frequency of transcriptional signatures described for S-Tip and D-Tip subpopulations in the retina^47^. No clear correlation is detected between retina and cardiac tip cell subpopulations.

**C.** List of tip cell marker genes described as down- or up-regulated in cardiac *Pdgfb*-expressing ECs upon 24 hours *Dll4* deletion^16^.

**D.** Feature marker plots of downregulated markers at E12 in the integrated UMAP.

**E.** Module score for significantly downregulated markers upon Dll4 loss shows enrichment in the CoEC V/Preartery cluster.

**F.** Feature marker plots of upregulated markers at E12 in the integrated UMAP.

**G.** Module score for significantly upregulated markers upon Dll4 loss shows enrichment in the CoEC I/Capillary-to-vein cluster.


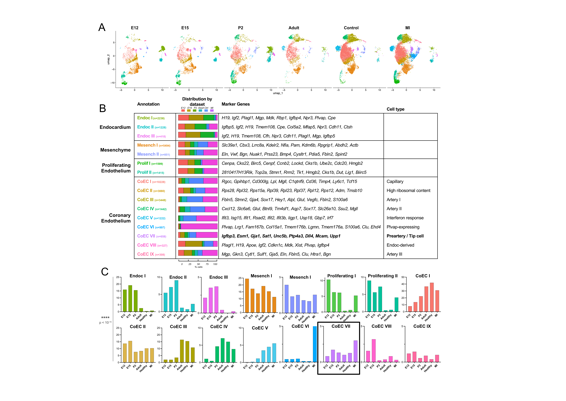


**Figure S12. Cluster annotation and distribution of E12, E15, P2, Adult, Control and MI integrated dataset.**

**A.** UMAP plot with annotated cluster split by dataset of origin.

**B.** Annotations and cell number of every cluster (first row), with their distribution throughout the different developmental stages (second row), top 10 marker genes (third row) and the assigned cell type.

**C**. Percentage of every cluster in each dataset, normalized by total cell number. CoEC VII / Preartery clusters proportion is increased in response to myocardial infarction. P value by χ2 test.

**
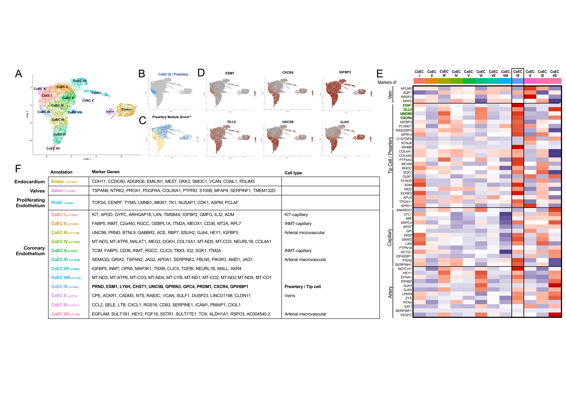
**

**Figure S13. Prearterial cell population with tip cell signature is detected in human embryos.**

**A.** UMAP plot with annotated cluster split by dataset of origin.

**B.** UMAP plot showing CoEC IX cluster highlighted in blue.

**C.** Module score of previously described prearterial marker genes^11^. CoEC IX shows a high score for this set of genes.

**D.** Feature marker plot of prearterial and tip cell markers (*Esm1, Cxcr4, Igfbp3, Dll4, Unc5b* ) and arterial marker (*Gja5*).

**E.** Heatmap of a wide list of known venous, tip cell, capillary and arterial markers in all coronary ECs clusters (CoEC I to CoEC IX).

**F.** Annotations and cell number of every cluster (first row), with their distribution throughout the different developmental stages (second row), top 10 marker genes (third row) and the assigned cell type.

**Supplementary tables’ legends**

**Table S1. Source data of figures 1D, 3G, 3H, 4C, 5D, S2C, S4G, S5F, S5G, S7B, S7D, S7E, S9B, S12B, S12C, S13**

**Table S2. Complete list of differentially expressed genes of figures 1D, S11A, S13F**

**Supplementary videos’ legends**

**Movie S1. Supplemental to Figure S5A.**

3-dimensional rendering of a E11.5 *PdgfbCreERT;R26mTmG* heart. GFP (green), Endom+VeCadh (magenta).

**Movie S2. Supplemental to Figure S5A.**

3-dimensional rendering of a E12.5 *PdgfbCreERT;R26mTmG* heart. GFP (green), Endom+VeCadh (magenta).

**Movie S3. Supplemental to Figure S5A.**

3-dimensional rendering of a E13.5 *PdgfbCreERT;R26mTmG* heart. GFP (green), Endom+VeCadh (magenta).

**Movie S4. Supplemental to Figure S5A.**

3-dimensional rendering of a E14.5 *PdgfbCreERT;R26mTmG* heart. GFP (green), Endom+VeCadh (magenta).

**Movie S5. Supplemental to Figure S5A.**

3-dimensional rendering of a E15.5 *PdgfbCreERT;R26mTmG* heart. GFP (green), Endom+VeCadh (magenta).

**Movie S6. Supplemental to Figure S5C.**

3-dimensional rendering of a E11.5 *PdgfbCreERT;R26mTmG* heart, magnification of the interventricular septum. GFP(green), Endom+VeCadh (magenta).

**Movie S7. Supplemental to Figure S5D.**

3-dimensional rendering of a E12.5 *PdgfbCreERT;R26mTmG* heart, magnification of the interventricular septum. GFP(green), Endom+VeCadh (magenta).

**Movie S8. Supplemental to Figure S5D1-D2.**

3-dimensional rendering of a E12.5 *PdgfbCreERT;R26mTmG* heart, magnification of movie 11. GFP(green), Endom+VeCadh (magenta).

**Movie S9. Supplemental to Figure S5E.**

3-dimensional rendering of a E15.5 *PdgfbCreERT;R26mTmG* heart, magnification of the ventral side of the right ventricle. GFP(green), Endom+VeCadh (magenta).

**MovieS10. Supplemental to Figure 2C.**

3-dimensional rendering of a E11.5 *PdgfbCreERT;R26mTmG* heart, magnification of the SV-derived plexus sprouting front. Segmented SV and SV-derived plexus (cyan), GFP+ within the endocardium (green).

**Movie S11. Supplemental to Figure S6.**

Time-lapse of a E12.5 *PdgfbCreERT;R26mTmG* heart, magnification of the right ventricle SV-derived plexus sprouting front. GFP (black).

**Movie S12 - Supplemental to Figure 2F**

3-dimensional rendering of a E11.5 *PdgfbCreERT;R26mTmG* heart, magnification of the atrioventricular canal. GFP (green), Endom+VeCadh (magenta).

**Movie S13. Supplemental to Figure S7A.**

3-dimensional rendering of a E11.5 *BmxCreERT;R26mTmG* heart. GFP (green), Endom+VeCadh (magenta).

**Movie S14. Supplemental to Figure S7C.**

3-dimensional rendering of a E15.0 *BmxCreERT;R26mTmG* heart. GFP+ cells within the coronary plexus(green), Endom+VeCadh (magenta).

**Movie S15. Supplemental to Figure S7F.**

3-dimensional rendering of a E15.0 heart. Veins and arteries are shown in blue and red respectively, and endocardium in gray.

**Movie S16. Supplemental to Figure 4D1.**

3-dimensional rendering of a E13.5 *Esm1CreERT;R26mTmG* heart, magnification of the sprouting front of the subepicardial plexus. GFP(green), Endom+VeCadh (magenta).

**Movie S17. Supplemental to Figure 4D2.**

3-dimensional rendering of a E13.5 *Esm1CreERT;R26mTmG* heart, magnification of the myocardial wall of LV. GFP(green), Endom+VeCadh (magenta).

**Movie S18. Supplemental to Figure 4D3.**

3-dimensional rendering of a E13.5 *Esm1CreERT;R26mTmG* heart, magnification of the subendocardial zone of LV. GFP(green), Endom+VeCadh (magenta).

**Movie S19. Supplemental to Figure 4D4.**

3-dimensional rendering of a E13.5 *Esm1CreERT;R26mTmG* heart, magnification of the interventricular septum. GFP(green), Endom+VeCadh (magenta).

**Movie S20. Supplemental to Figure 4D5.**

3-dimensional rendering of a E13.5 *Esm1CreERT;R26mTmG* heart, magnification of the aortic root. GFP(green), Endom+VeCadh (magenta).

**Movie S21. Supplemental to Figure 4F3.**

3-dimensional rendering of a E18.5 *Esm1CreERT;R26mTmG* heart, magnification subendocardial zone of the LV. GFP(green), Endom+VeCadh (magenta).
