# Supplementary material for: Intramyocardial Sprouting Tip Cells Specify Coronary Arterialization
Source: Circ Res. 2024 Aug 14;135(6):671–84. doi: 10.1161/CIRCRESAHA.124.324868 (PMC11361357; doi:10.1161/CIRCRESAHA.124.324868)
Supplement: Supplementary file 26 [file res-135-671-s026.pdf]

Major Resources Table

In order to allow validation and replication of experiments, all essential research materials listed in the Methods should be included in the Major Resources Table below. Authors are encouraged to use public repositories for protocols, data, code, and other materials and provide persistent identifiers and/or links to repositories when available. Authors may add or delete rows as needed.

Animals (in vivo studies)

Species	Vendor or Source	Background Strain	Sex	Persistent ID / URL
Mouse	Charles River	C57BL/6J	Female and male	C57BL/6J https://www.criver.com/products-services/find-model/c57bl6j-mice-jax-strain?region=3661
Mouse	Claxton et al., 2008.	*PdgfbCreERT* (C57BL/6J background)	Female and male	PdgfbCreERT2 (Tg(Pdgfb-icre/ERT2,-EGFP)1Frut) https://www.informatics.jax.org/allele/MGI:3793852
Mouse	Ehling et al., 2013	*BmxCreERT* (C57BL/6J background)	Female and male	Tg(Bmx-cre/ERT2)1Rha https://www.informatics.jax.org/allele/MGI:5513853
Mouse	Rocha et al., 2014	*Esm1CreERT* (C57BL/6J background)	Female and male	Tg(Esm1-cre/ERT2)1Rha N/A
Mouse	Muzumdar et al., 2007	R26mTmG (C57BL/6J background)	Female and male	Gt(ROSA)26Sortm4(ACTB-tdTomato,-EGFP)Luo/J https://www.jax.org/strain/007576

Genetically Modified Animals

	Species	Vendor or Source	Background Strain	Other Information	Persistent ID / URL
**Parent - Male**					
**Parent - Female**					

Antibodies

Target antigen	Vendor or Source	Catalog #	Working concentration	Lot # (preferred but not required)	Persistent ID / URL
aSMA-Cy3	Sigma	C6198	1:100		
CD31-APC	Thermo Fisher	17-0311-80	1:100		
CD45-PE	BD Biosciences	553081	1:100		
Cxcr4	Abcam	ab124824	1:100		
Donkey anti-Rabbit IgG - Alexa Fluor647	Invitrogen	A-31573	1:200		
Donkey anti-Rabbit IgG-Alexa Fluor568	Invitrogen	A-10042	1:200		
Endomucin	Santa Cruz	sc-65495	1:100		
ERa	Santa Cruz	sc-543	1:100		
GFP	Abcam	ab13970	1:100		
ICAM2	BD Pharmigen	553326	1:100		
Unc5b	Cell Signaling	13851	1:100		
VeCadherin	BD Biosciences	555289	1:100		
Donkey Anti-Chicken IgG-Alexa Fluor488	Jackson Immunoresearch,	703-545-155	1:200		

DNA/cDNA Clones

Clone Name	Sequence	Source / Repository	Persistent ID / URL

DOI [to be added]

Cultured Cells

Name	Vendor or Source	Sex (F, M, or unknown)	Persistent ID / URL

Data & Code Availability

Description	Source / Repository	Persistent ID / URL
Single-cell RNAseq raw data. (CD31+/CD45- cardiac endothelial fractions of C57Bl6J mouse hearts at different stages: E12, E15, P2 and 8 weeks-old adult animals)	Gene Expression Omnibus	GSE223266

Other

Description	Source / Repository	Persistent ID / URL
BSA	Fischer Scientific	BP1600-100
Collagenase II	Worthington	LS004176
Collagenase/Dispase	Sigma-Aldrich	11097113001
Dead cell removal kit	Miltenyi	130-090-101
Debris removal solution	Miltenyi	130-109-398
DiBenzyl Ether	Sigma-Aldrich	108014
Dicholoromethane	Sigma-Aldrich	270997
DMEM/F12	Thermo Scientific	11320
DMSO	Sigma-Aldrich	D4540
Donkey Serum	Jackson Immunoresearch	017-000-121
EBM2 media	Lonza	00190860
FBS	Merk F4135	F4135
Glass-bottomed culture dish	MatTek	P35G-1.5-14-C
Glycine	Sigma-Aldrich	G8898
Heparin Sodium Salt	AppliChem	A3004,0001
Hepes	Sigma-Aldrich	H3375
NP40	Thermo Scientific	85124
OCT embedding compound	Tissue-Tek	4583
Parafolmadehyde	Sigma-Aldrich	158127
Peanout Oil	Sigma-Aldrich	P2144
Penicillin/Streptomycin	Sigma-Aldrich	P4333
Red blood cells lysis step	Invitrogen	00-4333-57
Sodium Deoxycholate	Sigma-Aldrich	D6750
Sucrose	Sigma-Aldrich	S1888
Tamoxifen	Sigma-Aldrich	T5648
Tetrahidrofuran	Sigma-Aldrich	401757
Triton X-100	Sigma-Aldrich	T8787
Tween20	Sigma-Aldrich	P9416
Tyramide signal amplification kit	PerkinElmer	SAT704A001KT
Vectashield	Biozol	VEC-H-1200

DOI [to be added]

ARRIVE GUIDELINES
The ARRIVE guidelines (https://arriveguidelines.org/) are a checklist of recommendations to improve the reporting of research involving animals. Key elements of the study design should be included below to better enable readers to scrutinize the research adequately, evaluate its methodological rigor, and reproduce the methods or findings.
Study Design

Groups	Sex	Age	Number (prior to experiment)	Number (after termination)	Littermates (Yes/No)	Other description
Group 1 (Control)						
Group 2						
Add more if needed						

Sample Size: No statistical methods were used to predetermine sample size. Sample size were chosen based on experience while complying with animal welfare and ethic permit constraints. Sample size for each experiment (n) is provided in figure legends.

Inclusion Criteria: Animals with the right genotype and correct stage were included.

Exclusion Criteria: Data were not excluded from study.

Randomization: For imaging, no sample randomization was carried out as it was not relevant to our correlational study. For single-cell RNA sequencing, batch-effect was assessed by grouping cells by sex of donor for every stage. No differences were found in composition nor distribution of annotated cell types.

Blinding: No blinding was carried out as as it was not relevant to our study.

DOI [to be added]
